# BAP1 and USP1 cooperate to regulate FANCD2 stability and cell proliferation in mesothelioma cells

**DOI:** 10.1038/s41419-026-08818-7

**Published:** 2026-05-02

**Authors:** Koya Suzuki, Shinichi Kiyonari, Jo Nishino, Miki Takahashi, Yuna Kato, Miki Amano, Anna Ogiso, Tomohiro Akashi, Tohru Maeda, Norio Kaneda, Takashi Miida, Yutaka Kondo, Kenji Kadomatsu, Mamoru Kato, Koji Aoyama, Hiroshi Murakami, Yoshitaka Sekido, Yuko Murakami-Tonami

**Affiliations:** 1 Department of Pharmacology, Teikyo University School of Medicine, Itabashi-ku, Japan; 2 Advanced Comprehensive Research Organization, Teikyo University, Itabashi-ku, Japan; 3 Laboratory of Cancer Molecular Genetics, Tokyo University of Technology Graduate School of Bionics, Computer and Media Sciences, Hachioji, Japan; 4 Department of Biochemistry, Kitasato University School of Medicine, Sagamihara, Japan; 5 Division of Bioinformatics, Research Institute, National Cancer Center Japan, Tokyo, Japan; 6 Laboratory of Analytical Neurobiology, Faculty of Pharmacy, Meijo University, Nagoya, Japan; 7 Division of Cancer Biology, Aichi Cancer Center Research Institute, Nagoya, Japan; 8 Department of Integrative Cellular Informatics, Nagoya University Graduate School of Medicine, Nagoya, Japan; 9 College of Pharmacy, Kinjo Gakuin University, Nagoya, Japan; 10 Department of Clinical Laboratory Technology, Faculty of Medical Science, Juntendo University, Urayasu, Japan; 11 Division of Cancer Biology, Nagoya University Graduate School of Medicine, Nagoya, Japan; 12 Institute for Glyco-core Research (iGCORE), Nagoya University, Nagoya, Japan; 13 Department of Biological Science, Faculty of Science and Engineering, Chuo University, Bunkyo-ku, Japan; 14 Division of Molecular and Cellular Oncology, Nagoya University Graduate School of Medicine, Nagoya, Japan

**Keywords:** Mesothelioma, Cancer genetics

## Abstract

BRCA1-associated protein 1 (BAP1) is frequently inactivated in pleural mesothelioma and functions as a tumor suppressor through its deubiquitinating activity. In this study, we investigated the context-dependent interplay between BAP1 and ubiquitin-specific protease 1 (USP1) in mesothelioma cells, focusing on their roles in regulating FANCD2, cell proliferation, and DNA damage responses. Genetic suppression of USP1 selectively inhibited cell proliferation in BAP1-deficient mesothelioma cells, whereas reintroduction of wild-type BAP1 rescued this growth defect; notably, a catalytically inactive BAP1 mutant failed to do so, indicating that BAP1 deubiquitinase activity is required for this compensation. In contrast, depletion of FANCD2 suppressed cell proliferation irrespective of BAP1 status, underscoring the essential role of FANCD2 in mesothelioma cell survival. Although both BAP1 and USP1 were capable of deubiquitinating FANCD2 in vitro, USP1 suppression in mesothelioma cells did not provide clear biochemical evidence of altered FANCD2 ubiquitination. Instead, USP1 knockdown was associated with reduced FANCD2 transcript and protein levels, without markedly affecting FANCD2 mRNA stability. At the cellular level, USP1 depletion impaired FANCD2 focus formation and reduced its colocalization with γ-H2AX in BAP1-deficient cells, consistent with defective DNA damage signaling. Despite these changes, homologous recombination (HR) efficiency was largely preserved, whereas non-homologous end joining activity was modestly increased upon USP1 suppression. Consistent with these in vitro findings, USP1 knockdown suppressed tumor growth in an intrathoracic xenograft model. Collectively, our results indicate that BAP1 and USP1 appear to regulate FANCD2 through distinct, context-dependent mechanisms, with USP1 primarily influencing FANCD2 expression and BAP1 modulating FANCD2 function at the post-translational level. Together, these findings identify USP1 as a context-dependent therapeutic vulnerability in BAP1-deficient mesothelioma and support a working model in which USP1-dependent maintenance of FANCD2 function becomes critical in the absence of functional BAP1.

## Introduction

Mesothelioma is a highly aggressive cancer with poor prognosis, primarily associated with asbestos exposure. Despite advances in surgery, chemotherapy, and immunotherapy, patient survival remains dismal, with a median survival of approximately 1 year after diagnosis [1, 2]. The genomic landscape of mesothelioma is characterized by frequent alterations in tumor suppressor genes, with BRCA1-associated protein 1 (BAP1) being one of the most mutated genes [3–5]. *BAP1* encodes a nuclear deubiquitinating enzyme (DUB) involved in multiple cellular processes, including chromatin remodeling, transcriptional regulation, cell cycle control and DNA damage repair [6]. Germline mutations in *BAP1* are associated with a hereditary cancer syndrome, predisposing individuals to mesothelioma, uveal melanoma, and renal cell carcinoma [7–9]. Despite these insights, the precise molecular mechanisms by which *BAP1* deficiency contributes to tumorigenesis remain incompletely understood.

One potential link between BAP1 and cancer development is its involvement in DNA damage repair, including the Fanconi anemia (FA) pathway, although this connection remains underexplored. The FA pathway is essential for maintaining genomic stability by repairing DNA interstrand crosslinks and is frequently disrupted in various malignancies [10]. A key event in this pathway is the monoubiquitination of FANCD2, which facilitates its recruitment to DNA damage sites, promoting lesion bypass and repair [11]. This process is tightly regulated by the E3 ubiquitin ligase FANCL and counteracted by the deubiquitinating enzyme USP1, ensuring controlled activation of the FA pathway [12, 13]. Beyond FANCD2, USP1 deubiquitinates other key proteins involved in DNA repair, including FANCI, PCNA, and FANCC, implicating it as a central regulator of genome integrity [14–16]. Although direct evidence for USP1 deubiquitinating FANCC is lacking, FANCC mutations are well-established causes of Fanconi anemia and associated with increased cancer risk [17]. Given that BAP1-deficient cells exhibit defective DNA repair, it remains unclear how BAP1 and USP1 functionally interact in the FA pathway and whether their interplay could serve as a therapeutic target in mesothelioma.

A promising therapeutic approach in DNA repair-deficient cancers is synthetic lethality, a concept where the simultaneous loss of two genes results in cell death, whereas the loss of either gene alone is tolerated. This strategy has been successfully exploited in BRCA1/2-mutant cancers through PARP inhibitors, which block DNA repair, leading to tumor-specific cell death [18]. Since then, synthetic lethal genes for many cancer-causing genetic mutations have been reported [18–20]. In the context of mesothelioma, focal adhesion kinase (FAK) inhibitors were reported to have a synthetic lethal phenotype for mutations in NF2, one of the causative genes. However, despite an international phase II trial, the expected response rate was not achieved, and the trial was terminated in September 2015 [21–23]. Subsequently, it was reported that BAP1 mutations and class 1 HDAC (histone deacetylase) inhibitors show synthetic lethality [24, 25], and that LATS2 mutations and suppression of SMG6 expression produce a synthetic lethal phenotype in vitro and in vivo [26].

In this study, we investigate the functional relationship between BAP1 and USP1 in mesothelioma and explore USP1 inhibition as a potential synthetic lethal strategy in BAP1-deficient tumors [27]. Additionally, using tumor-bearing mouse models, we demonstrated that this phenotype is also observed in vivo. Understanding this interaction could not only provide insights into BAP1’s role in DNA repair but also identify novel therapeutic vulnerabilities in mesothelioma, paving the way for more effective treatments for this devastating disease.

## Materials and methods

### Mice

NOD/Shi-scid IL-2RγKOnull (NOG) mice were obtained from the Central Institute for Experimental Animals [28] (CIEA, Kawasaki, Japan). Mice were housed in the Juntendo University Graduate School of Medicine under specific pathogen-free conditions, on controlled 12 h light/dark cycles, and food and water ad libitum.

### Cell lines and cell culture

The human mesothelioma cell lines (NCI-H28, NCI-H226, NCI-H2373 and NCI-H2452) were purchased from ATCC. ACC-MESO-1 was originally established and characterized by Dr. Sekido [29], and is currently available from the Riken Bioresource Research Center (Tsukuba, Ibaraki, Japan); its BAP1 status has since been examined, including replication from our group [30]. H28 Venus, H28-BAP1, H28-BAP1(C91S), and H2452-BAP1 cells were generated in this study. Briefly, H28 Venus cells were generated by infecting H28 cells with a CSII-CMV-IRES-Venus vector-derived lentivirus, followed by sorting of Venus-positive cells using FACS Aria. BAP1 was amplified by PCR from pCold-His-BAP1 WT (kindly provided by Dr. Tomohiko Ohta, Dr. Hiroyuki Nishikawa) [31], and BAP1 C91S was amplified from pMMP-puro-BAP1(C91S) (kindly provided by Dr. Yuichi Machida) [32]. The functional properties of the C91S mutant have been described previously [33]. The amplified DNA fragments were cloned into pDONR (Invitrogen) and subsequently transferred into CSII-CMV-gateway-IRES-Venus (RIKEN Bioresource Center) using the Gateway® system (Invitrogen). Each cell line was infected with a lentivirus expressing BAP1 or BAP1(C91S), followed by sorting of Venus-positive cells using FACS Aria. All cell lines were cultured in RPMI-1640 medium supplemented with 10% fetal bovine serum (FBS) at 37 °C in a humidified incubator with 5% CO_2_. All cell lines were confirmed to be mycoplasma-free and were identified using short-tandem repeat analysis or a single nucleotide polymorphism array, as appropriate.

The shRNA plasmids and the expression plasmids used are listed in Table 1.

**Table 1:** Plasmids.

	Plasmid	Company	Source (Catalog No. or Reference)
Lentiviral vector	psPAX2	RIKEN Bioresorce Center	
	pMD2.G	RIKEN Bioresorce Center	
	pLKO.1-puro-shNT	Addgene	#109012
	shUSP1#45	Horizon (OpenBiosystems)	TRCN0000004045
	shUSP1#46	Horizon (OpenBiosystems)	TRCN0000004046
	shFANCD2#40	Horizon (OpenBiosystems)	TRCN0000082840
	shFANCD2#42	Horizon (OpenBiosystems)	TRCN0000082842
	shBAP1 #5090	Horizon (OpenBiosystems)	TRCN0000435090
	shBAP1 #7828	Horizon (OpenBiosystems)	TRCN0000417828
Expression vector	CSII-CMV-BAP1-IRES-Venus	in this study	
	CSII-CMV-BAP1 C91S-IRES-Venus	in this study	
	pET15-USP1	from Dr. Masuda,Dr. Masutani	
	pCold-His-BAP1	from Dr. Ohta, Dr. Nishikawa	*Cancer Research* **69**(1):111–119 (2009). DOI: 10.1158/0008-5472.CAN-08-3355
	pCold-His-BAP1 C91S	from Dr. Ohta, Dr. Nishikawa	*Cancer Research* **69**(1):111–119 (2009). DOI: 10.1158/0008-5472.CAN-08-3355
	pCBASce	from Dr. Jasin	*Genes & Development* **12**(24):3831-3842 (1998). DOI: 10.1101/gad.12.24.3831

### Cell viability assay

H28-Venus, H28-BAP1, and H28-BAP1(C91S) cells were plated at 2000 cells per well in 96-well plates and incubated for 24 h. Each well was treated with 10 µM MC2050 (Merck, NJ, USA; Cat. No. SML0213) or 400 µM 3-ABA (Merck, NJ, USA; Cat. No. 165350). After 72 h of treatment, cell viability and viability curves were analyzed using the Cell Counting Kit-8 (CCK-8; Dojindo, Kumamoto, Japan; Cat. No. CK04) according to the manufacturer’s instructions.

H28-Venus, H28-BAP1, and H28-BAP1(C91S) cells were also plated at 20,000 cells per well in 6-well plates and incubated for 24 h. The cells were then infected with lentivirus (non-target, shUSP1#45, shUSP1#46, shFANCD2#40, shFANCD2#42) in the presence of 4 µg/ml polybrene (Merck, NJ, USA; Cat. No. H9268). At 0, 48, 120, and 144 h post-transduction, the medium was replaced with 1 ml of 200 µM AlamarBlue reagent (Tokyo Chemical Industry, Tokyo, Japan; Cat. No. R0203), and the cells were incubated for 1 h [34]. Subsequently, 100 µl of the AlamarBlue solution from each well was transferred into 96-well plates. Absorbance was measured at 570 nm with 630 nm as a reference wavelength using a microplate reader. The average blank control was subtracted from all values. The absorbance at 0 h was set to 1, and relative cell viability at each time point was calculated.

### Reverse transcription and quantitative PCR

Total RNA was isolated using ISOSPIN Cells and Tissue RNA (FUJIFILM Wako Pure Chemical Corporation; Cat. No. 314-08211) or ISOGEN (Nippon Gene, Tokyo, Japan; Cat. No. 319-90211). cDNA was synthesized using ReverTraAce (TOYOBO, Osaka, Japan; Cat. No. TRT-101) according to the manufacturer’s instructions. Quantitative real-time PCR was performed using a KAPA SYBR Fast qPCR Kit (NIPPON Genetics, Tokyo, Japan; Cat. No. KK4602) and either the 7900HT Fast Real-Time PCR System or QuantStudio 3 (Applied Biosystems, Tokyo, Japan). Primer sequences are listed in Table 2.

**Table 2:** List of primers.

gene	forward	reverse
USP1	5′-GGACTTGGGGAAGTGTGAAA-3′	5′-AGCAACGGGTCCTTAATACC-3′
FANCD2	5′-GGACCTCACCACCAAGATCA-3′	5′-TCGGAGGCTTGAAAGGACAT-3′
GAPDH	5′-ATCATCCCTGCCTCTACTGG-3′	5′-CCCTCCGACGCCTGCTTCAC-3′
β-actin	5′-AGAAAATCTGGCACCACACC-3′	5′-AGAGGCGTACAGGGATAGCA-3′
MYC	5′-CCTACCCTCTCAACGACAG-3′	5′-CTCTGACCTTTTGCCAGGAG-3′
FANCD2 Intron5	5′-GAGGGAGAGCACAGTTGTTTC-3′	5′-CAAGGGAGAGGTAGAGATTTG-3′
FANCD2 Intron10	5′-GAGTCATACCTGCTTCCCATAG-3′	5′-ACTTCTCTTACTCCATCTTCCCAC-3′
RPLP0	5′-AGCCCAGAACACTGGTCTC-3′	5′-ACTCAGGATTTCAATGGTGCC-3′

### Protein purification

The pCold-His-BAP1 and pCold-His-BAP1 C91S expression plasmids (kindly provided by Dr. Ohta and Dr. Nishikawa) [31] were transformed into BL21 (DE3) Escherichia coli cells. Bacteria were cultured in LB medium at 37 °C until the optical density at 600 nm reached 0.5. The cultures were then cooled on ice to reduce the temperature to 15 °C, and IPTG was added to a final concentration of 400 μM to induce protein expression. After incubation for 24 h at 15 °C, bacteria were harvested and lysed by sonication in lysis buffer (20 mM Tris-HCl, pH 8.0, 0.5 M NaCl, 10 mM imidazole, 1% Triton X-100, 1 mM PMSF, 1 mM DTT, 10 mM benzamidine, 25 μg/mL leupeptin, 25 μg/mL antipain, and 10 μg/mL trypsin inhibitor). Cell lysates were clarified by centrifugation at 48,000 × *g* for 30 min at 4 °C, and the supernatants were incubated with Ni-NTA agarose (FUJIFILM Wako, Osaka, Japan; Cat. No. 143-09763) for 2 h at 4 °C with rotation. The resin was washed twice with lysis buffer and once with PBS, and bound proteins were eluted with 500 mM imidazole.

### Deubiquitination assay

Deubiquitination was assessed using Ub-FANCD2-FP (UbiQ, Amsterdam, Netherlands, Cat. No. UbiQ-029) following the manufacturer’s instruction [35]. In brief, purified proteins (BAP1 and BAP1 C91S) and recombinant USP1 (Recombinant Human His6-USP1, CF (R&D Systems, Minneapolis, MN, USA; Cat. No. E-564-050) were added at various concentrations into the wells of a 96-well clear bottom black plate. After Ub-FANCD2-FP diluted with the reaction buffer was dispensed into each well, fluorescence polarization (FP) (excitation at 531 nm, emission at 579 nm) was recorded every 30 s for 30 min using FlexStation3 (Molecular Devices, Tokyo, Japan). pMAX indicated the FP value of FANCD2-FP with buffer, and pMIN indicated the FP value of 5-TAMRA (5-carboxytetramethylrhodamine; CosmoBio, Tokyo, Japan, Cat. No. ABD-363-10).

### Western blotting

Cells were harvested either in Cell Lytic™ M (Sigma-Aldrich, St. Louis, MO, USA) or lysed from frozen cell pellets in RIPA buffer supplemented with 0.1% SDS and protease inhibitors, followed by sonication on ice. Lysates were cleared by centrifugation (≥12,000 × *g*, 10 min, 4 °C), and supernatants were mixed with 2× Laemmli sample buffer and heated at 95 °C for 5 min. Equal amounts of protein were resolved by SDS–PAGE using 10% gels for BAP1 and β-actin, and 6% gels for FANCD2 and Vinculin. Proteins were transferred to PVDF membranes under standard wet transfer conditions. Membranes were blocked with either 5% (w/v) non-fat dry milk in TBS-T (20 mM Tris-HCl, 150 mM NaCl, 0.1% Tween-20) or 3% (w/v) BSA in TBS, then incubated with primary antibodies as listed in Table 3 overnight at 4 °C. After washing in TBS-T, membranes were incubated with appropriate HRP-conjugated secondary antibodies for 1 h at room temperature, washed, and developed with Luminata™ Forte Western HRP Substrate (Merck Millipore, Darmstadt, Germany). Chemiluminescence was captured using either the GE ImageQuant™ LAS-800 (IQ800) system or Lumina Graph I (ATTO, WSE-6100H-CSP).

**Table 3:** List of antibodies.

Epitope	Company	Catalog number	
BAP1 (1G8)	Novus Biologicals	NB110-60521	
β-Actin	CST	#3700	
FANCD2	CST	#16323	for WB
FANCD2	Funakoshi	NB100-182	for IF
Vinculin Santa Cruz sc-73614			
γ-H2AX	Merck	05-636	
Ki-67	abcam	ab15580	
Anti-rabbit IgG, HRP-linked antibody	CST	#7074	
Anti-mouse IgG, HRP-linked antibody	CST	#7076	
Alexa Flour 647 goat anti-mouse IgG	Thermo Fisher Scientific	A21236	
Alexa Flour 594 goat anti-rabbit IgG	Thermo Fisher Scientific	A11037	

### Transcriptional inhibition assay

For transcriptional inhibition experiments, cells were treated with actinomycin D (ActD; 5 μg/mL, Wako, Tokyo, Japan, 018-21264) to inhibit transcription, and total RNA was collected at the indicated time points (0, 2, 4, 6, 8, 12, 18, and 24 h after ActD addition). The expression levels of FANCD2 and MYC were quantified by RT–qPCR using the ΔΔCt method, as described above.

### TCGA-MESO data analysis

Mesothelioma cases from TCGA (TCGA-MESO) were obtained from cBioPortal (study: meso_tcga_pan_can_atlas_2018) and analyzed [36–38]. mRNA expression z-scores (RNA-seq V2 RSEM; as provided by cBioPortal) and somatic mutation data were used. BAP1 status was defined using somatic short variants only (wild type: no mutation or missense; mutant: truncating or likely loss-of-function variants).

The association between USP1 and FANCD2 expression was evaluated using Spearman’s rank correlation. A multivariable linear regression model was fitted with FANCD2 expression as the outcome and USP1 expression and BAP1 mutation status as covariates (FANCD2 ~ USP1 + BAP1_mut). For survival analysis, patients were stratified into USP1-high and USP1-low groups using the cohort-wide median expression as the cutoff. Kaplan–Meier survival curves were generated separately within the BAP1 wild-type and BAP1-mutant subgroups, and differences were assessed by the log-rank test.

### GTEx normal lung data analysis

For comparison with normal tissue, GTEx lung RNA-seq data (gene_tpm_v11_lung.gct) were analyzed [39]. Expression values (TPM) were transformed as log2 (TPM + 1), and correlations between gene expression levels were assessed using Spearman’s rank correlation.

### Homologous recombination assay

HR efficiency was assessed using U2OS cells stably harboring the DR-GFP reporter construct (DR-U2OS cells; kindly provided by Dr. Jasin) [40]. For gene knockdown or overexpression experiments, cells were transduced with lentiviral vectors encoding the respective shRNA or cDNA, followed by selection to establish stable expression. To induce a site-specific double-strand break, cells were transiently transfected with the pCBASce plasmid, which expresses the I-SceI endonuclease, using FuGENE® Transfection Reagent (Promega, Madison, WI, USA) according to the manufacturer’s instructions. At 48 h after transfection, cells were harvested, resuspended in PBS, and analyzed by flow cytometry using a BD FACSCalibur™ System (BD Biosciences, San Jose, CA, USA). The proportion of GFP-positive cells was quantified as a measure of HR frequency, and data were processed using CellQuest™ Software (BD Biosciences, San Jose, CA, USA).

### NHEJ assay

NHEJ activity was measured using the H1299dA3-1 reporter cell line, a derivative of H1299 cells harboring a GFP-based substrate with inverted I-SceI sites (H1299dA3-1 cells; kindly provided by Dr. Kohno) [41, 42]. Gene overexpression or knockdown was achieved by lentiviral transduction, and double-strand breaks were introduced by transient transfection of an I-SceI expression vector using FuGENE® Transfection Reagent (Promega, Madison, WI, USA). 48 h later, GFP-positive cells were quantified on a BD FACSCalibur™ System (BD Biosciences, San Jose, CA, USA) and analyzed with CellQuest™ Software (BD Biosciences, San Jose, CA, USA), and NHEJ efficiency was expressed relative to control.

### Immunofluorescence staining

For immunostaining, cells, which were seeded on the cover glass, were fixed by the ice-cold methanol at 4 °C for 10 min or by 4% paraformaldehyde at RT for 10 min followed by permeabilization using 0.05% Triton X-100. The samples were then blocked with phosphate buffer saline (PBS) containing 1% bovine serum albumin (BSA) at room temperature (RT) for 30 min. Then, the samples were stained with primary antibodies listed in Table 3 overnight at 4 °C. After three washes with PBS, the samples were stained with fluorescently labeled secondary antibodies listed in Table 3 with 1 μg/ml DAPI for 30 min at RT. After washing, samples were embedded in fluorescence mounting medium (Dako Japan, Tokyo, Japan; Cat. No. S3023).

### Microscopy

Immunofluorescent images were captured with a BZ-X810 (Keyence, Osaka, Japan) and FV3000 (Olympus, Tokyo, Japan). Fluorescence images were analyzed using ImageJ software (National Institutes of Health, Bethesda, MD, USA).

### In vivo experiments

Luciferase-H226 cells were plated on the 10 cm culture dish (As ONE, Osaka, Japan). After 24 h incubation, cells were infected with lentivirus (non-target, shUSP1#45, or shUSP1#46) using 4 μg/ml polybrene for 24 h. Then these cells were gently detached and counted. Six-week-old female NOG mice were anesthetized using medetomidine–midazolam–butorphanol. 1 × 10^6^ each cell was injected into the right thoracic cavity [43]. Each mouse was intraperitoneally injected with 5 μmol/mouse D-luciferin (abcam, Cambridge, UK, Cat No. ab145164). After 15 min, to assess tumor development, luminescence was quantified using the IVIS^TM^ imaging system (Summit Pharmaceuticals, Tokyo, Japan).

### Tissue preparation

All animals were euthanized by carbon dioxide inhalation, and tumor and lung tissues were collected. Tissues were washed with ice-cold phosphate-buffered saline (PBS) to remove residual blood and fixed in 10% neutral buffered formalin for paraffin embedding. After fixation, samples were processed using CT-Pro20 (GenoStaff) and embedded in paraffin. Paraffin sections were deparaffinized in xylene (room temperature, 3 min, twice) and rehydrated through a graded ethanol series (100%, 95%, 90%, 80%, 70%, and 50%; 1 min each). Hematoxylin and eosin (H&E) staining and immunohistochemistry were performed according to standard protocols. For immunofluorescence analysis of frozen sections, tumor tissues were embedded in Optimal Cutting Temperature (OCT) compound (SAKURA Finetek, Tokyo, Japan; Cat. No. 4583) and stored at −80 °C. Sections (5 μm) were prepared using a cryostat.

Frozen sections were fixed in methanol at −20 °C for 10 min or in 4% paraformaldehyde in PBS, followed by permeabilization with 0.01% Triton X-100 for 10 min. Sections were blocked with 1% BSA in PBS for 30 min at room temperature and incubated overnight at 4 °C with primary antibodies listed in Table 3, diluted in 1% BSA/PBS. After washing three times with PBS, sections were incubated for 30 min at room temperature with fluorophore-conjugated secondary antibodies diluted in 1% BSA/PBS, together with 1 μg/mL 4′,6-diamidino-2-phenylindole (DAPI; BioLegend Japan, Tokyo, Japan; Cat. No. 1351303). Sections were washed three times with PBS and once with distilled water and mounted using fluorescence mounting medium.

### Statistical analysis

All experimental data are expressed as the mean ± SD, unless otherwise indicated. Comparisons between two groups were performed using Student’s *t* test, and differences were considered statistically significant at *p* < 0.05. Comparisons among more than three groups were analyzed using one-way ANOVA on ranks, followed by the Tukey–Kramer post hoc test using Statcel4 software (OMS Publishing, Tokyo, Japan). For in vivo experiments, data are presented as the mean ± SEM. Statistical methods for analyses of TCGA-MESO and GTEx normal lung data are described earlier in the “Materials and methods” section.

### Ethics approval and consent to participate

All animal experiments were performed in accordance with protocols approved by the Animal Studies Committee of Juntendo University (the Ethics Committee for Animals at Juntendo University, Approval No. 1401). Recombinant DNA experiments were approved by the Committee for Recombinant DNA Experiments at Juntendo University (Approval No. DNA30-69) and at Tokyo University of Technology (Approval Nos. 20-BS-01-067 and 20-BS-02-068). Establishment of cell lines was approved by the Ethics Committee of the Aichi Cancer Center (Approval No. 19-12), and written informed consent was obtained from the patients.

## Results

### USP1 knockdown suppresses cell proliferation in BAP1-deficient mesothelioma cells but not in BAP1-expressed cells

We conducted a genome-wide shRNA library screening to identify synthetic lethal interactions with BAP1 mutations, a key genetic alteration in mesothelioma, and identified several candidate genes (data not shown). Among these, we focused on USP1.

**Fig. 1:**
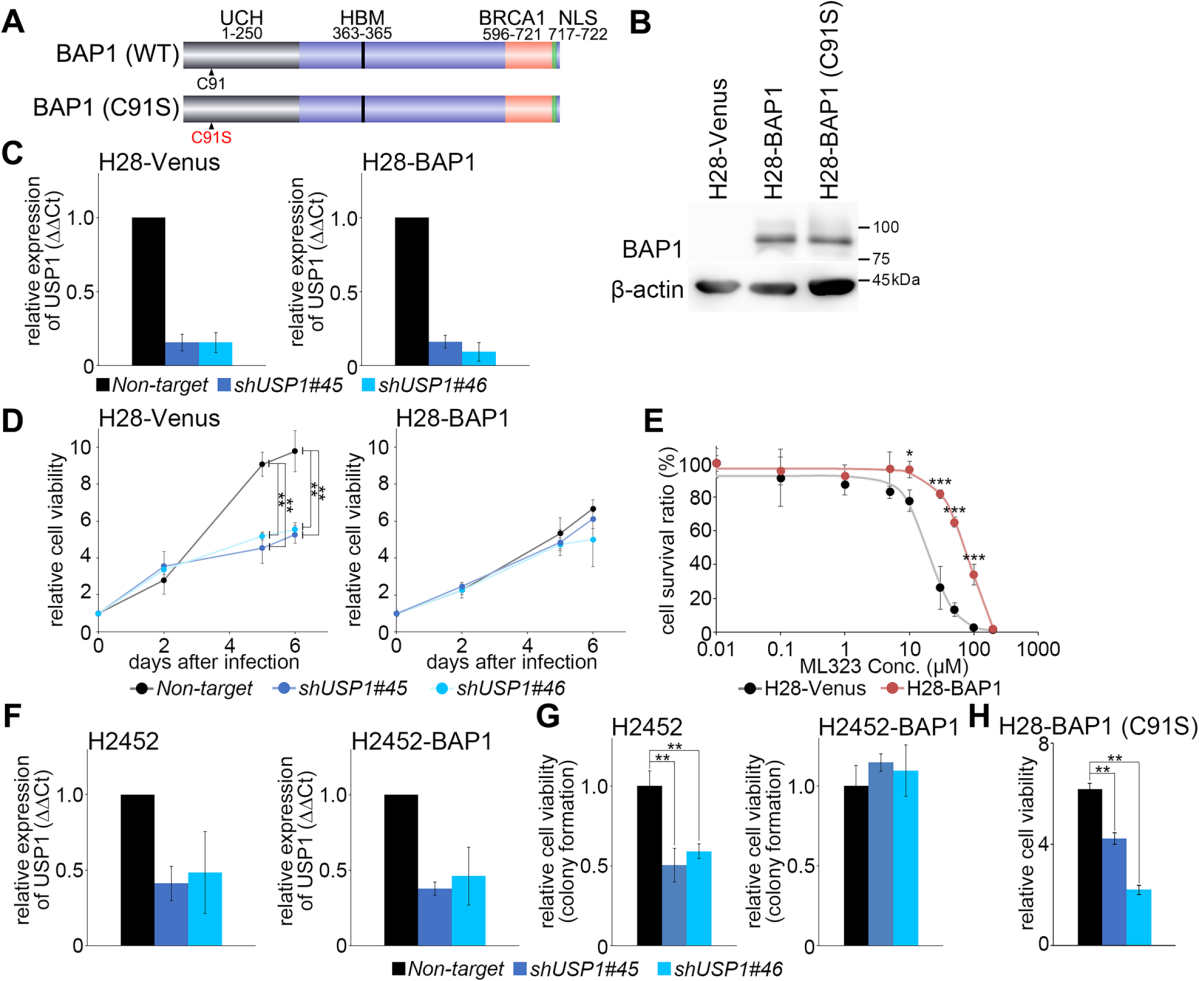
Suppression of USP1 expression inhibited cell proliferation in BAP1-deficient mesothelioma cell lines. **A** Schematic representation of BAP1 constructs used in this study. UCH—ubiquitin carboxyl hydrolase domain (1–250); HBM—HCF1 binding domain (365–385); BRCA1 binding region (596–721) and NLS—nuclear localization signals (656–661 and 717–722). **B** Protein expression levels of BAP1 in H28 cells stably expressing Venus as a control (H28-Venus), H28 cells overexpressing wild-type BAP1 (H28-BAP1), and H28 cells overexpressing the catalytically inactivated mutant BAP1 (C91S) [H28-BAP1 (C91S)]. β-actin served as a loading control. **C** The efficiency of USP1 knockdown in the H28-Venus and H28-BAP1 cells was assessed by RT-qPCR. **D** Cell viability of USP1-knockdowned H28-Venus and H28-BAP1 cells. The indicated shRNA was introduced, and cell counts were assessed at 0-, 2-, 5-, and 6-days post-infection using AlamarBlue staining. Values, normalized to 0-day cells, are expressed as means ± SD from 3 independent experiments. Statistical significance compared with non-target shRNA infected cells (Tukey-Kramer test): **P < 0.01. **E** Cell viability was assessed in H28-Venus and H28-BAP1 cells treated for 72 h with the indicated concentrations of the USP1–UAF1 inhibitor ML323 (0.01, 0.1, 1, 5, 10, 30, 50, 100, and 200 μM; R&D Systems, Minneapolis, MN, USA). Cell viability was measured using the Cell Counting Kit-8 (CCK-8; Dojindo, Kumamoto, Japan) according to the manufacturer’s instructions, and values were normalized to DMSO-treated controls. Data are presented as mean ± SD. Statistical significance between H28-Venus and H28-BAP1 cells at each concentration was determined using Student’s *t* test (*P < 0.05, ***P < 0.001). **F** USP1 knockdown efficiency during the colony formation assay shown in (**G**). **G** Anchorage-independent growth assessed by colony formation assays in BAP1-deficient H2452 cells and wild-type BAP1-overexpressing H2452 cells (H2452-BAP1) following USP1 suppression. Cells were infected with the indicated lentiviral shRNAs and selected with puromycin for 3 days, harvested, and replated. After 2 weeks, colonies were fixed and stained. Colony numbers were quantified and normalized to non-target-treated cells. Statistical significance was determined using the Tukey–Kramer test (**P < 0.01). **H** Proliferation of USP1-knockdown H28-BAP1(C91S) cells was assessed by counting cells after 6days after infection. Ratio of cell count relative to non-target control is expressed as the mean ± SD (*n* = 3). Statistical significance compared with non-target shRNA infected cells (Tukey-Kramer test): **P < 0.01.

To validate this finding, using the BAP1-deficient NCI-H28 cell line, we prepared H28 cells expressing Venus control (H28-Venus), H28 cells overexpressing wild-type BAP1 (H28-BAP1), and H28 cells overexpressing the catalytically inactive BAP1 mutant with C91S (H28-BAP1(C91S)) (Fig. 1A, B). We then suppressed USP1 expression using shRNA (Fig. 1C) and evaluated the effects on cell proliferation. USP1 knockdown significantly inhibited proliferation in H28-Venus cells (Fig. 1D, left), whereas this effect was not observed in H28-BAP1 cells (Fig. 1D, right). Consistently, pharmacological inhibition of USP1 with ML323 (Fig. 1E) revealed increased sensitivity in H28-Venus cells, whereas this sensitivity was rescued in H28-BAP1 cells, in line with prior reports of ML323 as a selective USP1–UAF1 inhibitor [44, 45].

We next examined whether the growth-inhibitory phenotype specifically induced by shUSP1 knockdown could be recapitulated in other mesothelioma cell lines. Specifically, we analyzed the effects of BAP1 expression in H226 and H2452 cells, both of which harbor BAP1 mutations (Fig. S1A). In these BAP1-mutated cells, USP1 suppression significantly reduced proliferation, consistent with the results in H28 cells (Fig. S1B). By contrast, USP1 knockdown had little effect on proliferation in BAP1–wild-type cell lines (ACC-MESO-1 [29] and H2373) or in H2452 cells engineered to express wild-type BAP1 (H2452-BAP1) (Fig. S1C).

To further assess this phenotype under anchorage-independent conditions, we performed colony formation assays using H2452 cells and H2452 cells stably expressing wild-type BAP1 (H2452-BAP1). BAP1 expression in H2452-BAP1 cells is shown in Fig. S1A, and USP1 knockdown efficiency is shown in Fig. 1F. Consistent with the results obtained in adherent cultures, USP1 suppression significantly reduced colony formation in BAP1-deficient H2452 cells, whereas no such effect was observed in H2452-BAP1 cells (Fig. 1G).

Finally, we investigated whether the deubiquitinating (DUB) activity of BAP1 is required for this synthetic lethal phenotype. To this end, we assessed proliferation in H28-BAP1(C91S) cells. Unlike wild-type BAP1 reconstitution, expression of the DUB-inactive BAP1 mutant (C91S) failed to rescue the proliferation defects induced by USP1 knockdown (Fig. 1H).

Collectively, these findings demonstrate that USP1 suppression selectively impairs both anchorage-dependent proliferation (Fig. 1D and Fig. S1B) and anchorage-independent growth (Fig. 1G) in BAP1-mutated cells, highlighting the requirement of BAP1 deubiquitinating activity for this synthetic lethal phenotype.

### BAP1 deubiquitinates FANCD2 in vitro

To elucidate the molecular mechanism underlying the inhibition of cell proliferation upon USP1 suppression in BAP1-mutated cells, we considered the well-established roles of BAP1 and USP1 in DNA repair, as well as their shared function as deubiquitinating enzymes. Given this, we focused on the involvement of deubiquitinating enzyme activity in DNA repair mechanisms.

To investigate this, we examined whether BAP1 can directly deubiquitinate FANCD2 in vitro, as FANCD2 is a well-characterized substrate of USP1-mediated deubiquitination [46, 47]. To this end, we performed a deubiquitination assay using a class II FP DUB activity probe (Ub-FANCD2-FP), which was constructed based on the FANCD2 peptide sequence 557–565 containing the monoubiquitination site Lys561. As a positive control, we first examined USP1, a well-established FANCD2 deubiquitinating enzyme, to validate the assay system (Fig. S2A), in which a decrease in millipolarization (mP) reflects cleavage of ubiquitin from the FANCD2 peptide and thereby indicates deubiquitination activity. USP1 promoted a nearly concentration-dependent acceleration of FANCD2 deubiquitination at 0.312, 0.625, 1.25, and 2.5 μM; beyond 0.625 μM, the reaction rate reached a plateau, and complete deubiquitination was ultimately observed at all concentrations tested. Because this assay employed a minimal peptide-based substrate, the observed USP1 activity should be interpreted as evidence of intrinsic catalytic competence under simplified conditions, whereas efficient deubiquitination of full-length FANCD2 in cells typically requires the USP1–UAF1 complex.

**Fig. 2:**
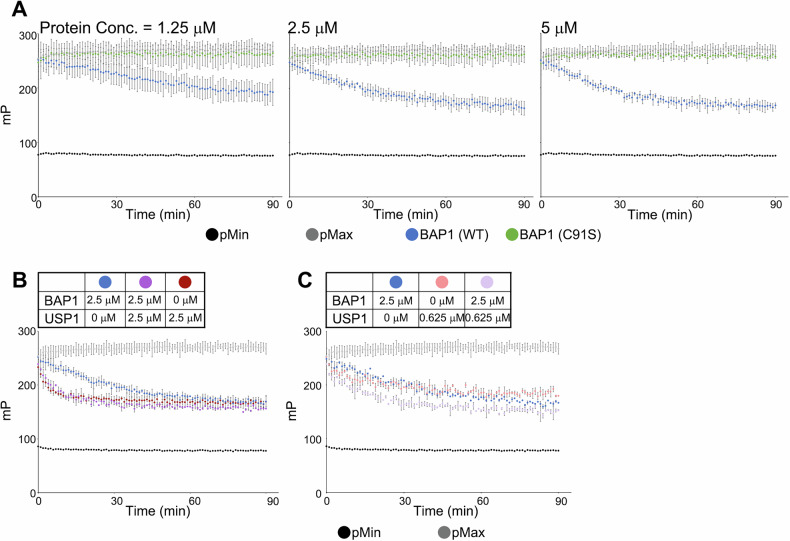
In the absence of USP1, BAP1 deubiquitinates FANCD2 in vitro. **A** Reaction time course for the FANCD2-derived FP probe in the presence of BAP1 (WT) (blue circles) or BAP1 (C91S) (green circles) at 1.25, 2.5, and 5 μM. pMax (gray circles) was detected using Ub-FANCD2-FP without catalysis and pMin (black circles) indicated the mP of TAMRA peptide. **B** Reaction time course for the FANCD2-derived FP probe in the presence of 2.5 μM BAP1 (blue circles), 2.5 μM USP1 (red circles), or both (purple circles). pMax (gray circles) was detected using Ub-FANCD2-FP without catalysis and pMin (black circles) indicated the mP of TAMRA peptide. **C** Reaction time course for the FANCD2-derived FP probe in the presence of 2.5 μM BAP1 (blue circles), 0.625 μM USP1 (orange circle), or both (purple circles). pMax (gray circle) was detected using Ub-FANCD2-FP without catalysis and pMin (black circles) indicated the mP of TAMRA peptide.

We then tested purified wild-type BAP1 protein or a catalytically inactive BAP1 mutant (C91S) in the same system and monitored FP. As shown in Fig. 2A, wild-type BAP1 induced a concentration-dependent decrease in FP (1.25–5 μM), demonstrating its capacity to deubiquitinate FANCD2 (Fig. 2A, blue circles). In contrast, the C91S mutant had no effect, similar to the blank control (Fig. 2A, green circles).

Next, we directly compared the activities of BAP1 and USP1 at an equal concentration (2.5 μM). After 60 min, both enzymes reduced FP to a similar extent (Fig. 2B, blue and red circles), indicating comparable overall activity. However, USP1 reached a plateau more rapidly than BAP1 and retained activity at lower concentrations (Fig. 2B, blue and red circles). Furthermore, co-addition of BAP1 and USP1 (Fig. 2B, purple circles) produced a fluorescence reduction comparable to USP1 alone, suggesting that USP1 predominates under these conditions.

To further evaluate relative potency, we repeated the assay using a four-fold lower concentration of USP1 than BAP1 (Fig. 2C and Fig. S2B). Notably, USP1 displayed nearly equivalent activity despite its lower concentration.

Collectively, these findings validate USP1 as a robust positive control, confirm that BAP1 possesses intrinsic deubiquitinating activity toward FANCD2, and demonstrate that USP1 exhibits higher efficiency and specificity under the tested conditions. These results prompted us to investigate whether the deubiquitinating activity of BAP1 toward FANCD2 observed in vitro also translates into functional consequences in cells, particularly in the context of DNA damage responses.

### BAP1 and USP1 regulate FANCD2 expression and cell proliferation

**Fig. 3:**
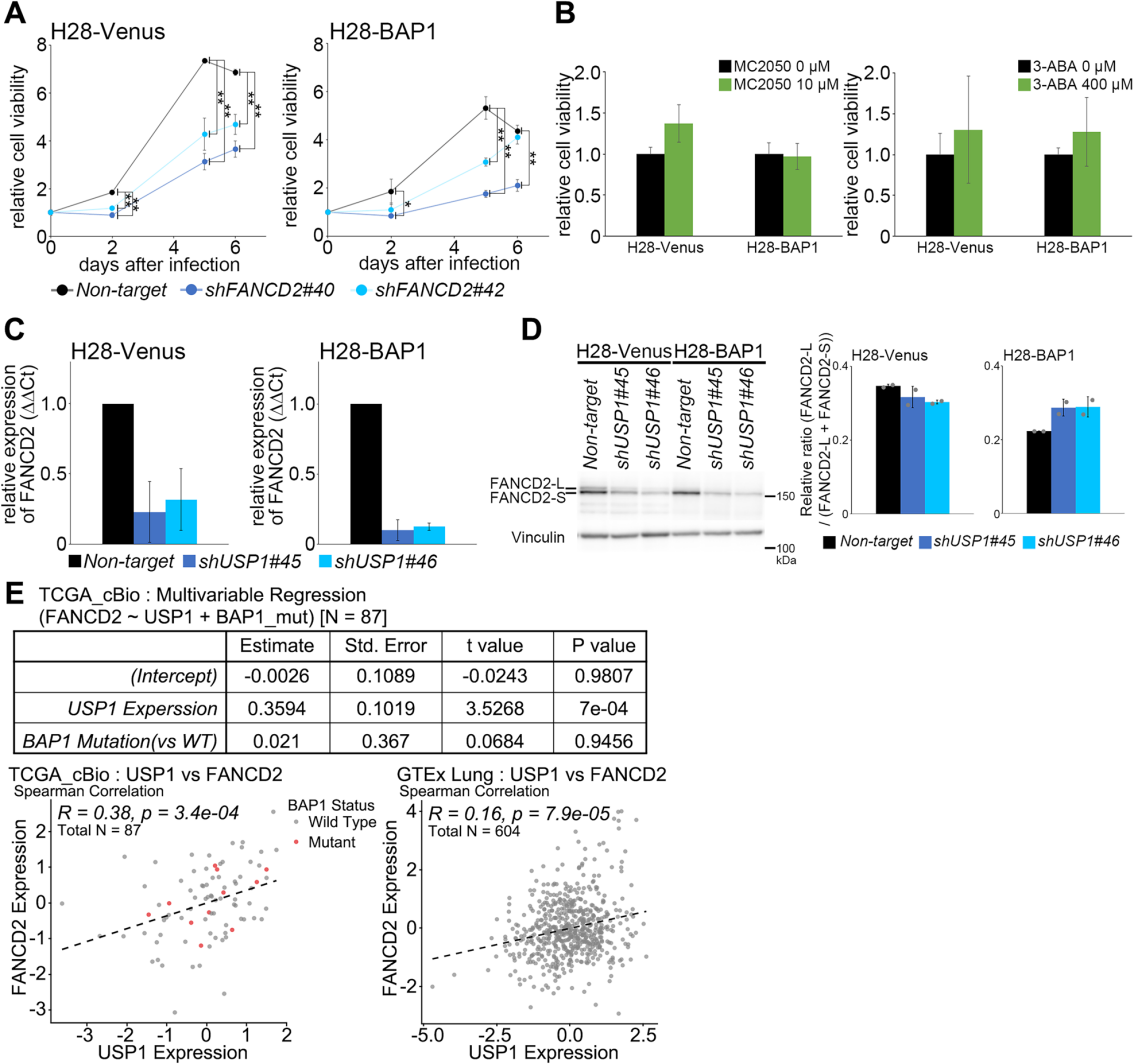
Regulation of FANCD2 levels and ubiquitination by BAP1 overexpression and USP1 knockdown and clinical correlates. **A** The cell viability of H28-Venus or H28-BAP1 cells. The indicated shRNA was introduced, and cell counts were assessed at 0-, 2-, 5-, and 6-days post-infection using AlamarBlue staining. Values, normalized to 0-day cells, are expressed as means ± SD from 3 independent experiments. Statistical significance compared with non-target shRNA infected cells (Tukey-Kramer test): *P < 0.05, **P < 0.01. **B** The cell viability of H28-Venus cells and H28-BAP1 cells treated with PARP1 inhibitors (MC2050, 3-ABA). The cell survival rates were assessed 72 h after PARP1 inhibitors treatment using the Alamar Blue assay, and the relative survival rates were calculated with the drug concentration at 0 mM set as 1.0. **C** The expression of FANCD2 by RT-qPCR after USP1 knockdown in H28-Venus and H28-BAP1 cells. RNAs were collected 48 h after infection with a virus expressing shUSP1. **D** Endogenous FANCD2 monoubiquitination was assessed by electrophoretic mobility shift (FANCD2-L: monoubiquitinated form; FANCD2-S: non-ubiquitinated form) following USP1 knockdown in H28-Venus and H28-BAP1 cells. Vinculin was used as a loading control. Cells were harvested 72 h after infection with shUSP1-expressing virus. Quantification of the FANCD2-L proportion was performed by calculating the ratio of FANCD2-L to total FANCD2 (FANCD2-L/ (FANCD2-L + FANCD2-S)). Bars represent the mean of two independent experiments, with individual data points shown. **E** Association between USP1 and FANCD2 mRNA expression in public datasets. Upper panel: Multivariable linear regression analysis of FANCD2 expression using USP1 expression and BAP1 mutation status as covariates in the TCGA mesothelioma cohort (TCGA-MESO; cBioPortal; *N* = 87). Lower panels: Correlation between USP1 and FANCD2 mRNA expression in the TCGA-MESO cohort (left; mRNA z-scores, *N* = 87) and in GTEx lung samples (right; log₂ [TPM + 1], *N* = 604). Spearman correlation coefficients (*R*) and corresponding *p* values are shown. In the TCGA-MESO plot, samples are colored according to BAP1 mutation status (wild-type vs mutant).

Next, we investigated the effect of FANCD2 knockdown on cell proliferation in BAP1-deficient cells. The results demonstrated that FANCD2 knockdown suppressed cell proliferation regardless of the presence or absence of wild-type BAP1 (Fig. 3A). These findings suggest that the synthetic lethal phenotype is primarily determined by the ubiquitination status of FANCD2, rather than by its overall protein abundance.

Given previous reports indicating that BAP1 interacts with BRCA1 and contributes to DNA repair [48], we examined whether BAP1-deficient cells exhibit sensitivity to PARP inhibitors. Using two different PARP inhibitors, we assessed the sensitivity of H28 cells. Previous studies have reported conflicting results regarding the impact of BAP1 alterations on sensitivity to PARP inhibitors, with some suggesting enhanced sensitivity in mesothelioma models [49, 50], while others found no clear association [51]. In line with the latter, our experiments showed that H28 cells harboring BAP1 mutations did not exhibit significant sensitivity to PARP inhibitors (Fig. 3B). If homologous recombination (HR) repair were impaired due to BAP1 deficiency, these cells would be expected to be sensitive to PARP1 inhibitors. However, our results suggest that alternative DNA repair pathways, such as non-homologous end joining (NHEJ), may be activated in BAP1-deficient cells to compensate for HR dysfunction. To further clarify the regulatory mechanisms involving FANCD2, we assessed its expression and ubiquitination status following USP1 knockdown. RT–qPCR analysis showed that USP1 knockdown reduced FANCD2 transcript levels (Fig. 3C). Given this reduction, we next examined whether it could be attributed to altered mRNA stability. To assess transcriptional inhibition efficiency, MYC mRNA, a short-lived transcript, was monitored following actinomycin D treatment. As shown in Fig. S3A (left panel), MYC mRNA levels rapidly declined in both control and USP1-knockdown cells, confirming effective inhibition of transcription. Under the same conditions, FANCD2 mRNA exhibited comparable decay kinetics between control and USP1-depleted cells (Fig. S3A, right panel), indicating that USP1 suppression does not markedly affect FANCD2 mRNA stability. We then examined whether FANCD2 downregulation occurs upstream of mRNA stability by analyzing FANCD2 pre-mRNA levels. Using two independent intronic primer sets, FANCD2 pre-mRNA levels were consistently reduced upon USP1 knockdown (Fig. S3B, C), supporting regulation at the transcriptional level rather than through altered mRNA decay. At the protein level, BAP1 overexpression in H28 cells led to a modest reduction in FANCD2 expression, whereas USP1 knockdown caused a more pronounced decrease in total FANCD2 protein levels (Fig. 3D). Assessment of the FANCD2 mobility shift (FANCD2-L/S) did not demonstrate a robust or uniform change in the monoubiquitinated fraction under these conditions. Therefore, while the data support regulation of FANCD2 abundance by USP1 and BAP1, they do not provide definitive biochemical evidence for direct modulation of FANCD2 ubiquitination in cells. To assess whether this relationship is also observed in human tumors, we analyzed publicly available mesothelioma datasets. In the TCGA malignant pleural mesothelioma cohort (TCGA-MESO), FANCD2 mRNA expression showed an association with USP1 expression when analyzed using a multivariable linear regression model incorporating BAP1 mutation status as a covariate (Fig. 3E, upper panel). Consistent with this, a positive correlation between USP1 and FANCD2 mRNA expression was observed in TCGA-MESO samples, with distinct patterns depending on BAP1 mutation status (Fig. 3E, lower left panel). Consistent with this, analysis of GTEx normal lung samples revealed a correlation between USP1 and FANCD2 expression in non-malignant tissue (Fig. 3E, lower right panel), providing contextual support for a coordinated regulation of these genes. Together, these findings suggest that BAP1 and USP1 regulate FANCD2 expression and function through distinct mechanisms.

### USP1 and BAP1 influence DNA repair pathway regulation and FANCD2 localization

To gain mechanistic insight into how USP1 knockdown may influence DNA repair pathway regulation, we employed established HR and NHEJ reporter assays.

**Fig. 4:**
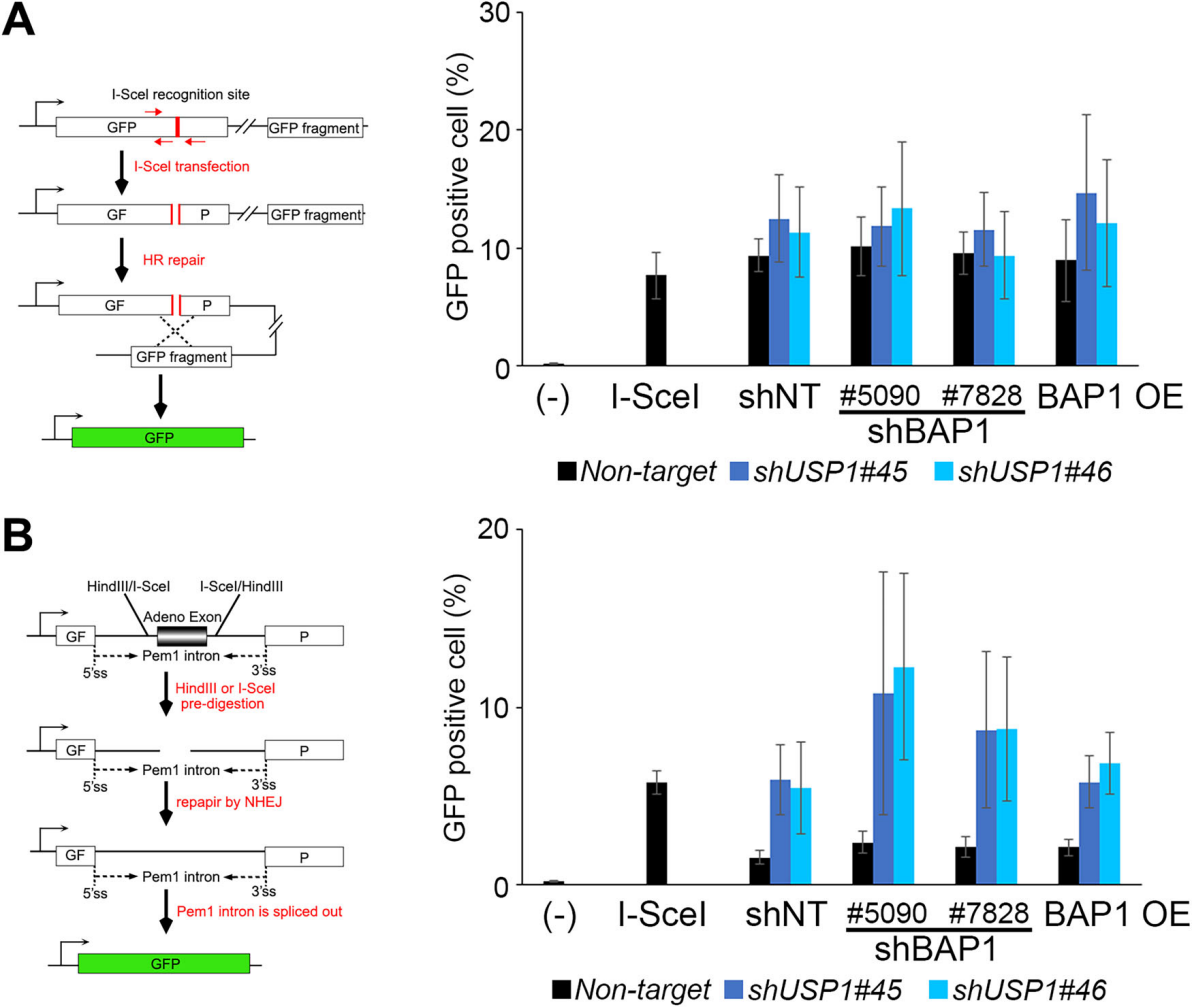
Effects of BAP1 expression and USP1 knockdown on HR and NHEJ activities. **A** HR assay after altering BAP1 and USP1 expression levels in U2OS cells stably harboring the DR-GFP reporter construct (DR-U2OS cells). **B** NHEJ assay after altering BAP1 and USP1 expression levels in H1299dA3-1 cells.

As shown in Fig. 4A, neither BAP1 overexpression nor USP1 knockdown led to significant changes in HR activity in the reporter system, indicating that neither protein directly affects canonical HR efficiency in this experimental context. In contrast, the NHEJ reporter assay (Fig. 4B) showed a modest increase in NHEJ activity upon USP1 knockdown. Because HR activity remained unchanged, this effect is unlikely to reflect a compensatory shift from HR to NHEJ. Rather, these results suggest that USP1 can influence the balance of DNA repair pathway usage in reporter-based systems, although the magnitude of the effect was limited. Consistent with this interpretation, BAP1-deficient mesothelioma cells did not exhibit synthetic lethality upon PARP inhibition (Fig. 3B), arguing against a major defect in canonical HR. Taken together, these findings indicate that the proliferation defects observed upon USP1 knockdown are unlikely to be explained by gross impairment of canonical HR or NHEJ activity, and instead point to altered regulation of FANCD2.

**Fig. 5:**
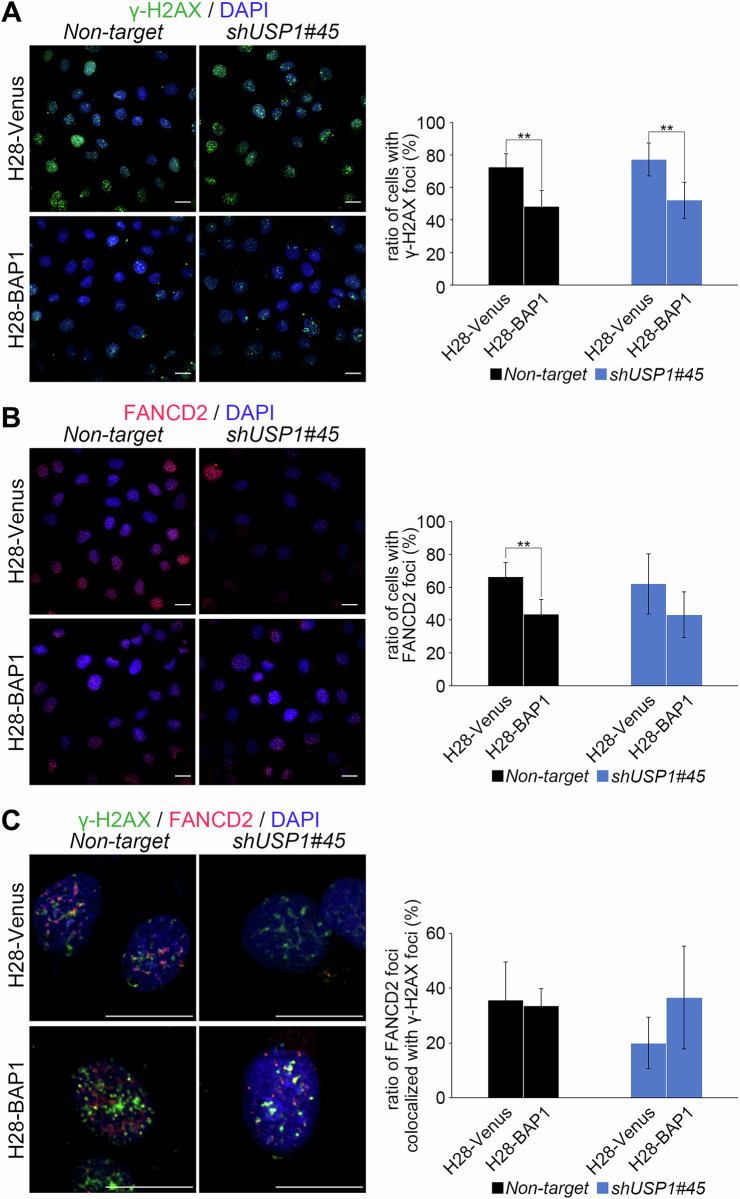
Regulation of DNA damage signaling and FANCD2 recruitment by BAP1 and USP1. **A** Immunofluorescence detection of γ-H2AX (green) with Alexa647 conjugated antibody in H28-Venus and H28-BAP1 cells after 72 h infected with each lentivirus. Nuclei were stained in blue with 4’,6-diamidino-2-phenylindole (DAPI). The γ-H2AX positive cells were counted with over 5 foci. γ-H2AX positive cell ratio was normalized total number of DAPI. Values are expressed as means ± SD. Statistical significance compared with H28-Venus and H28-BAP1 with non-target or shUSP1 was determined using Tukey-Kramer test: **P < 0.01. Scale bars indicated 20 μm. **B** Immunofluorescence detection of FANCD2 (red) with Alexa546 conjugated antibody. Nuclei were stained in blue with DAPI. The FANCD2 positive cells were counted as nuclei with red foci. FANCD2 positive cell ratio was normalized total number of DAPI. Data are presented as the means ± SD. Statistical significance compared with H28-Venus and H28-BAP1 at non-target or shUSP1 was determined using Tukey-Kramer test: **P < 0.01. Scale bars indicated 20 μm. **C** The ratio of co-localization of γ-H2AX foci and FANCD2 foci upon USP1 knockdown in H28 and H28-BAP1 cells. The ratio of FANCD2 foci colocalized with γ-H2AX was normalized the total number of γ-H2AX. Values are expressed as means ± SD. Scale bars indicated 20 μm.

Based on this possibility, we next examined the localization of FANCD2 at DNA damage sites. In H28-Venus cells expressing nontargeting shRNA (shNT), γ-H2AX foci, a marker of DNA damage, were readily observed (Fig. 5A). In H28-BAP1 cells, the number of γ-H2AX–positive cells was significantly reduced compared with H28-Venus cells under both shNT and shUSP1 conditions (Fig. 5A). In contrast, USP1 knockdown did not significantly alter the proportion of γ-H2AX–positive cells when compared with the corresponding shNT controls in either H28-Venus or H28-BAP1 cells (Fig. 5A). We then analyzed FANCD2 localization under these conditions. Distinct FANCD2 foci were observed in H28-Venus cells with either shNT or shUSP1, indicating that USP1 suppression alone did not alter FANCD2 focus formation (Fig. 5B). In contrast, BAP1 overexpression significantly reduced the number of FANCD2 foci, as shown by the comparisons between H28-Venus and H28-BAP1 cells under both shNT and shUSP1 conditions (Fig. 5B). Finally, colocalization analysis revealed that the overlap of γ-H2AX and FANCD2 foci was significantly decreased in USP1-knockdown H28 Venus cells (Fig. 5C).

While depletion of FANCD2 suppresses cell proliferation irrespective of BAP1 status, our data suggest that USP1 knockdown alters FANCD2 localization specifically in BAP1-deficient cells. This alteration may contribute to impaired FANCD2 function, which could in turn account for the observed proliferation defect upon USP1 knockdown only in BAP1-mutant cells, thereby providing a potential mechanistic basis for the synthetic lethal interaction between USP1 suppression and BAP1 deficiency.

### USP1 suppression in BAP1-deficient cells recapitulates growth suppression in vivo

To examine whether the growth-suppressive effect of USP1 knockdown observed in vitro is also evident in vivo, we performed intrathoracic xenograft experiments using H226 cells, which harbor a BAP1 mutation. Although the histological origin of H226 cells has been debated, this model was used as a supplementary in vivo system to assess the reproducibility of the observed phenotype.

**Fig. 6:**
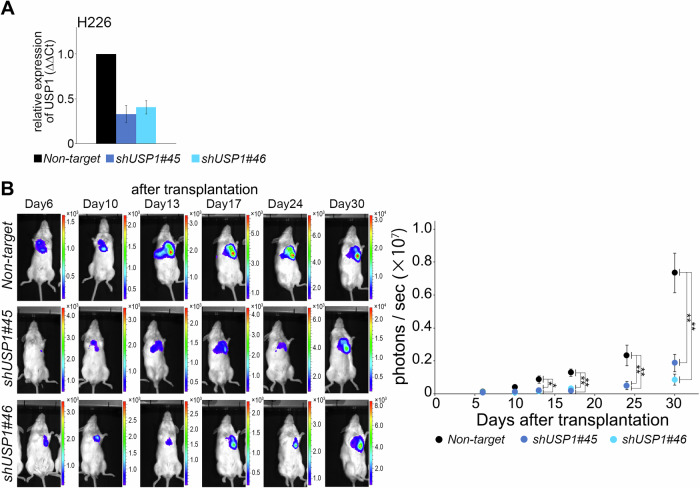
USP1 knockdown suppresses tumor growth of BAP1-mutated H226 cells in an intrathoracic xenograft model. **A** Validation of USP1 knockdown efficiency in H226 cells prior to transplantation, as determined by RT–qPCR. USP1 mRNA levels were normalized to GAPDH and expressed relative to cells transduced with non-targeting shRNA (non-target). **B** In vivo tumor growth of H226 cells transduced with non-target or two independent USP1-targeting shRNAs (shUSP1 #45 and #46) following intrathoracic transplantation into mice. Representative bioluminescence images acquired at the indicated time points after transplantation are shown (left). Quantification of total photon flux over time is presented on the right. Data are shown as mean ± SEM (non-target, *n* = 5; shUSP1 #45, *n* = 4; shUSP1 #46, *n* = 4). Statistical significance was assessed relative to non-target controls.

H226 cells transduced with non-targeting shRNA or two independent USP1-targeting shRNAs were transplanted into the mouse thoracic cavity, and tumor progression was monitored longitudinally by bioluminescence imaging. Efficient USP1 knockdown was confirmed prior to transplantation (Fig. 6A). As shown in Fig. 6B, USP1 suppression resulted in a marked reduction in tumor growth over time compared with control cells, and quantitative analysis of photon flux demonstrated a significant suppression of tumor burden in USP1-depleted tumors.

To further characterize the in vivo phenotype at the tissue level, histological analyses were performed on xenograft tumors (Supplementary Fig. S4). Hematoxylin and eosin staining revealed reduced tumor mass in USP1-knockdown xenografts, while immunofluorescence analyses showed decreased Ki-67–positive cells, indicating reduced proliferative capacity. In addition, FANCD2-positive nuclear foci were observed in tumor sections (Supplementary Fig. S4C), indicating that FANCD2 signals are present in vivo under these experimental conditions. Notably, FANCD2-positive foci appeared to show reduced colocalization with γH2AX in USP1-knockdown tumors compared with shNT controls (Supplementary Fig. S4D), suggesting impaired recruitment or retention of FANCD2 at sites of DNA damage in vivo. Together, these results indicate that USP1 suppression inhibits tumor growth in vivo and induces molecular and cellular changes consistent with impaired proliferation and altered DNA damage responses, in agreement with the growth-suppressive effects observed in BAP1-deficient cells in vitro.

### Clinical relevance of USP1 expression in BAP1-mutated malignant pleural mesothelioma

To explore the clinical relevance of the USP1–BAP1 axis in malignant pleural mesothelioma (MPM), we analyzed publicly available TCGA MPM datasets using cBioPortal. Patients were stratified based on BAP1 mutation status, excluding cases with BAP1 missense mutations to focus on tumors with likely loss-of-function alterations.

**Fig. 7:**
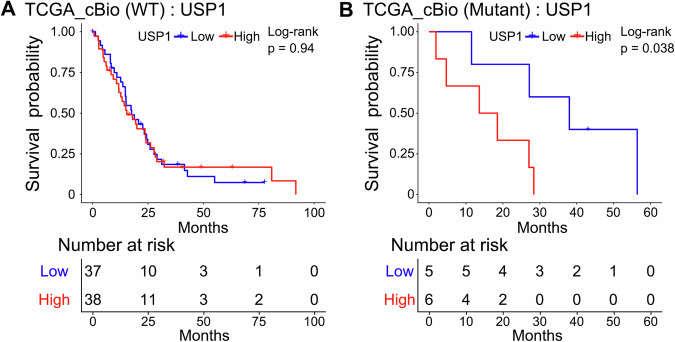
Prognostic impact of USP1 expression in TCGA malignant pleural mesothelioma (TCGA-MESO) cases stratified by BAP1 mutation status. **A** Kaplan–Meier analysis of overall survival in TCGA-MESO cases classified as BAP1 wild type, stratified by USP1 mRNA expression levels (high vs. low, dichotomized at the cohort median; *N* = 75). **B** Kaplan–Meier analysis of overall survival in TCGA-MESO cases classified as BAP1 mutant, stratified by USP1 mRNA expression levels (high vs. low, dichotomized at the cohort median; *N* = 11). TCGA-MESO patient data were analyzed using cBioPortal. BAP1 missense mutations were excluded to enrich for tumors with likely loss-of-function alterations. Numbers of patients at risk are shown below each plot.

We first examined the association between USP1 mRNA expression levels and overall survival in BAP1–wild-type MPM patients. Kaplan–Meier analysis revealed no significant difference in overall survival between patients with high versus low USP1 expression in this group (Fig. 7A; log-rank test, *p* = 0.94). In contrast, among BAP1-mutated MPM patients, lower USP1 mRNA expression was significantly associated with improved overall survival (Fig. 7B; log-rank test, *p* = 0.038). These results indicate that the prognostic relevance of USP1 expression is context-dependent and becomes apparent specifically in the setting of BAP1 deficiency.

## Discussion

**Fig. 8:**
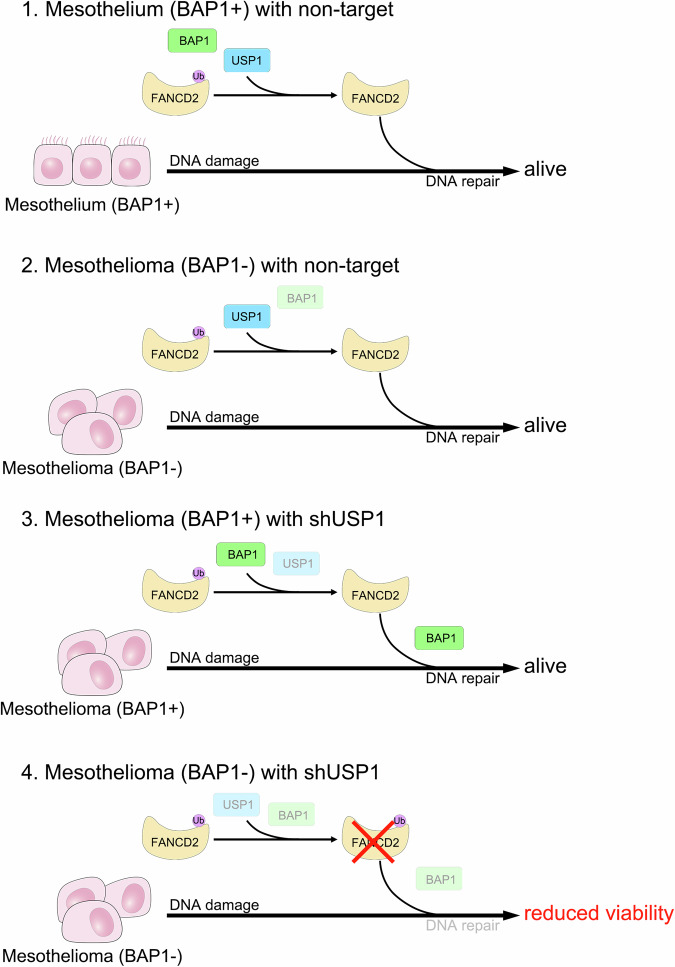
A context-dependent model of FANCD2 functional regulation by USP1 and BAP1 in mesothelioma cells. 1. In normal mesothelial cells, DNA damage induces activation of FANCD2-dependent genome maintenance pathways. FANCD2 function is regulated through coordinated mechanisms operating at multiple levels, with USP1 contributing to the maintenance of FANCD2 expression and BAP1 supporting aspects of FANCD2 regulation at the post-translational level. Together, these regulatory inputs enable efficient recruitment of FANCD2 to DNA damage sites, thereby promoting effective DNA damage responses and cellular homeostasis. 2. In mesothelioma cells harboring loss-of-function mutations in BAP1, FANCD2-dependent genome maintenance becomes increasingly reliant on USP1. Under these conditions, USP1 plays a critical role in sustaining FANCD2 expression and functional availability, which are essential for cancer cell survival. 3. Upon suppression of USP1, FANCD2 transcript and protein levels are reduced, and FANCD2 localization to γ-H2AX–marked DNA damage sites is impaired. Although ubiquitin immunoprecipitation–based assays were not performed, mobility shift analysis of FANCD2 (FANCD2-L/S) and changes in FANCD2 abundance were consistent with altered FANCD2 regulatory state. In BAP1-proficient cells, residual BAP1-dependent regulatory mechanisms partially compensate for USP1 loss, allowing cells to tolerate USP1 depletion. 4. In contrast, in BAP1-deficient mesothelioma cells, compensatory regulation by BAP1 is absent. As a result, USP1 suppression leads to impaired FANCD2-dependent DNA damage signaling, accumulation of DNA damage, and growth suppression. Because FANCD2 is essential for mesothelioma cell viability regardless of BAP1 status, these findings are consistent with a model in which partial functional impairment of FANCD2 becomes selectively detrimental in BAP1-deficient cells, thereby contributing to the synthetic lethal phenotype. This model integrates our in vitro and in vivo observations and highlights USP1 as a context-dependent therapeutic vulnerability in BAP1-deficient mesothelioma.

Our findings highlight context-specific roles of BAP1 and USP1 in regulating cell proliferation and DNA repair in mesothelioma cells. Genetic suppression of USP1 selectively inhibited proliferation in BAP1-deficient cells but not in BAP1-overexpressing cells, except when the overexpressed BAP1 lacked deubiquitinase activity. In contrast, depletion of FANCD2 suppressed cell growth irrespective of BAP1 status, indicating that FANCD2 function is essential for mesothelioma cell survival, whereas the synthetic lethal phenotype observed upon USP1 suppression is strongly dependent on the BAP1-deficient context. Based on these findings, we propose a context-dependent model in which USP1 becomes essential for maintaining FANCD2 function specifically in BAP1-deficient mesothelioma cells, leading to a synthetic lethal interaction upon USP1 suppression (Fig. 8).

Consistent with previous reports implicating BAP1 in HR–associated DNA repair, we examined whether BAP1-deficient mesothelioma cells exhibit sensitivity to PARP1 inhibition. However, BAP1-mutant H28 cells did not show increased sensitivity to PARP inhibitors, in line with studies reporting no clear association between BAP1 status and PARP inhibitor response. These findings suggest that BAP1 deficiency alone does not confer a classical HR-deficient phenotype and that alternative repair pathways, such as NHEJ, may compensate for HR impairment in this setting. Importantly, the growth-inhibitory effects of USP1 suppression were clearly distinct from those of PARP inhibition, underscoring that USP1 targeting represents an alternative vulnerability beyond canonical HR deficiency.

At the molecular level, USP1 knockdown reduced FANCD2 expression at both the mRNA and protein levels, whereas BAP1 overexpression led to only modest changes in FANCD2 protein abundance. Transcriptional inhibition experiments demonstrated comparable FANCD2 mRNA decay kinetics between control and USP1-depleted cells, excluding altered mRNA stability as the primary mechanism. In addition, FANCD2 pre-mRNA levels were consistently reduced upon USP1 suppression, supporting regulation at the transcriptional level. Together, these findings indicate that USP1 influences FANCD2 expression upstream of mRNA stability rather than through modulation of transcript decay. Despite the well-established role of USP1 as a deubiquitinase for FANCD2 in the canonical Fanconi anemia pathway, our study did not provide clear biochemical evidence that USP1 directly regulates FANCD2 ubiquitination in mesothelioma cells. Immunoblot analyses failed to detect consistent accumulation of ubiquitinated FANCD2 species upon USP1 knockdown, highlighting a potential divergence between canonical FA pathway regulation and the regulatory landscape in mesothelioma. Instead, immunofluorescence analyses revealed persistent γ-H2AX foci in BAP1-mutant cells, accompanied by reduced FANCD2 foci formation and diminished colocalization of FANCD2 with γ-H2AX following USP1 suppression. These defects are consistent with impaired FANCD2 recruitment to DNA damage sites and inefficient repair, ultimately leading to cell cycle arrest and growth inhibition.

Collectively, our data support a model in which BAP1 and USP1 regulate FANCD2 through distinct regulatory layers. USP1 primarily influences FANCD2 expression at the transcript level in BAP1-deficient contexts, whereas BAP1 modulates FANCD2 function at the post-translational level, potentially through its deubiquitinase activity. In BAP1-deficient cells, USP1 appears to function as the dominant regulator sustaining FANCD2-dependent DNA repair capacity, rendering these cells particularly vulnerable to USP1 suppression.

From a translational perspective, our findings underscore the therapeutic potential of targeting USP1 in mesothelioma harboring BAP1 loss-of-function alterations, a disease for which effective targeted therapies remain limited. Consistent with genetic knockdown experiments, pharmacological inhibition of USP1 using the small-molecule inhibitor ML323 selectively suppressed proliferation in BAP1-deficient cells, an effect that was rescued by BAP1 overexpression. Although ML323 demonstrated robust activity in vitro, our in vivo analyses remain preliminary, and further studies will be required to optimize dosing, scheduling, and combination strategies, as well as to assess specificity, potential off-target effects, and tolerability.

Several questions remain to be addressed. Identification of additional substrates of BAP1 and USP1, clarification of their direct molecular interactions, and deeper mechanistic analysis of FANCD2–γ-H2AX dynamics will further elucidate how DNA repair pathway balance dictates cell fate in mesothelioma. Moreover, given the heterogeneity of this disease, it will be essential to define which patient subsets derive the greatest benefit from USP1-targeted strategies.

Finally, analysis of TCGA malignant pleural mesothelioma datasets revealed that USP1 expression is associated with patient outcome specifically in the context of BAP1-deficient tumors, supporting the clinical relevance of the context-dependent functional interaction between USP1 and BAP1. Together, these findings establish USP1 as a promising therapeutic vulnerability in BAP1-deficient mesothelioma and provide a framework for future translational and clinical investigations.

## Supplementary information

Supplementary Figure Legend

Supplemental Figure 1

Supplemental Figure 2

Supplemental Figure 3

Supplemental Figure 4

Original Data

## Data Availability

All data and materials are available in the main text or the supplementary materials, or from the corresponding author upon reasonable request.
