# Supplementary material for: BAP1 and USP1 cooperate to regulate FANCD2 stability and cell proliferation in mesothelioma cells
Source: Cell Death Dis. 2026 May 2;17(1):583. doi: 10.1038/s41419-026-08818-7 (PMC13279938; doi:10.1038/s41419-026-08818-7)
Supplement: Supplementary file 1 — Supplementary Figure Legend [file 41419_2026_8818_MOESM1_ESM.docx]

**Figure S1 BAP1 status and the effects of USP1 knockdown on cell proliferation across mesothelioma cell lines**

(A) Immunoblot analysis of BAP1 expression in H2452, H2452-BAP1, and H226 cells. H2452 and H226 cells are BAP1-deficient, whereas H2452-BAP1 cells stably overexpress wild-type BAP1. β-Actin was used as a loading control.

(B) Effects of USP1 knockdown on cell viability in BAP1-deficient H226 and H2452 cells. Cell viability was measured 72 h after transduction using the Alamar Blue assay. USP1 knockdown significantly reduced viability compared with cells transduced with non-targeting shRNA.

(C) Effects of USP1 knockdown on cell viability in BAP1-proficient settings, including H2452-BAP1, ACC-MESO-1, and H2373 cells. Cell viability was measured 72 h after transduction using the Alamar Blue assay. In these BAP1–wild-type contexts, USP1 knockdown had little or no impact on viability compared with non-target.

Data represent mean ± SD from at least three independent experiments. Statistical significance compared with non-target shRNA infected cells (Tukey-Kramer test): *P < 0.05, **P < 0.01.

**Figure S2 Comparative deubiquitination activity of BAP1 and USP1 toward FANCD2 in vitro**(A) Validation of the fluorescence polarization (FP)–based deubiquitination assay using USP1 as a positive control. The FANCD2-derived FP probe was incubated with increasing concentrations of USP1 (0.312–2.5 μM: gradually deepening red circles), resulting in a concentration-dependent decrease in fluorescence polarization. pMax (grey circles) was detected using Ub-FANCD2-FP without catalysis and pMin (black circles) indicated the mP of TAMRA

(B) Comparison of BAP1 and USP1 activity at equal concentrations. FANCD2-derived FP probes were incubated with BAP1 or USP1 at 0.625 (yellow circles), 1.25 (green circles), or 2.5 μM (purple circles). Both enzymes reduced fluorescence polarization, although USP1 showed more rapid activity (left). Combined effects of BAP1 and USP1 at varying ratios. FANCD2-derived FP probes were incubated with BAP1 (fixed at 2.5 μM) together with USP1 at 0 (blue circle), 0.625, 1.25, or 2.5 μM (gradually darkening purple circles). The reduction in fluorescence polarization was comparable to USP1 alone, indicating that USP1 predominates under these conditions (right). pMax (grey circles) was detected using Ub-FANCD2-FP without catalysis and pMin (black circles) indicated the mP of TAMRA

**Figure S3** **Effect of transcriptional inhibition on MYC and FANCD2 mRNA expression.**

(A) MYC (left) and FANCD2 (right) mRNA levels in H28-Venus cells treated with actinomycin D (ActD) at the indicated time points, analyzed by quantitative RT–PCR to assess mRNA decay kinetics.

(B) FANCD2 pre-mRNA levels in H28-Venus and H28-BAP1 cells measured using intron 5–specific primers.

(C) FANCD2 pre-mRNA levels in H28-Venus and H28-BAP1 cells measured using intron 10–specific primers.

Expression levels were normalized to the reference gene and are shown relative to the corresponding non-target control.

**Figure S4 Histological and immunofluorescence analyses of H226 xenograft tumors following USP1 knockdown**

(A) Hematoxylin and eosin (H&E) staining of intrathoracic xenograft tumors derived from H226 cells transduced with non-targeting shRNA or USP1-targeting shRNAs (#45 and #46). Representative images of tumor and adjacent lung tissue are shown. Scale bars, 100 μm

(B) Immunofluorescence staining for Ki-67 (green) and DAPI (blue) in xenograft tumor sections, showing reduced proliferative activity in USP1-knockdown tumors. Scale bars, 20 μm

(C) Immunofluorescence staining for FANCD2 (red) and DAPI (blue) in xenograft tumors. Scale bars, 10 μm

(D) Immunofluorescence staining for γH2AX (green), FANCD2 (red), and DAPI (blue) in xenograft tumors. Scale bars, 10 μm
