# Supplementary figures and images for: BAP1 and USP1 cooperate to regulate FANCD2 stability and cell proliferation in mesothelioma cells
Source: Cell Death Dis. 2026 May 2;17(1):583. doi: 10.1038/s41419-026-08818-7 (PMC13279938; doi:10.1038/s41419-026-08818-7)

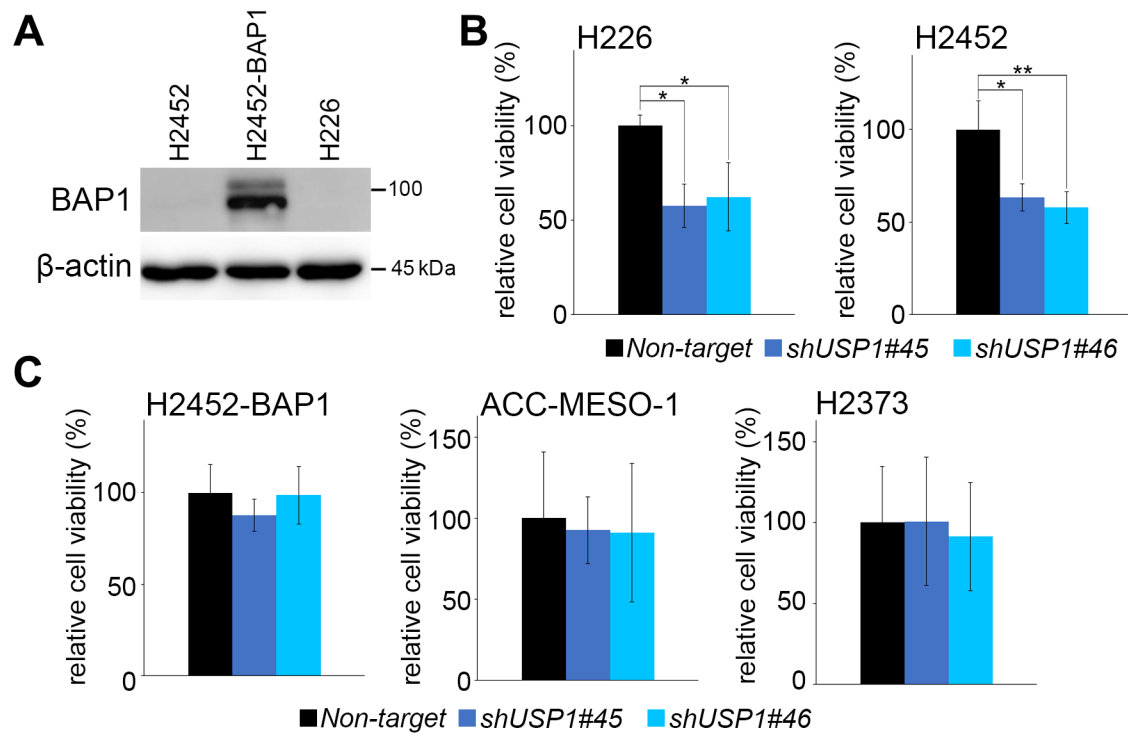

supplementary Figure 1 Suzuki K et al.

Supplement: Supplementary file 2 — Supplemental Figure 1 [file 41419_2026_8818_MOESM2_ESM.pdf]

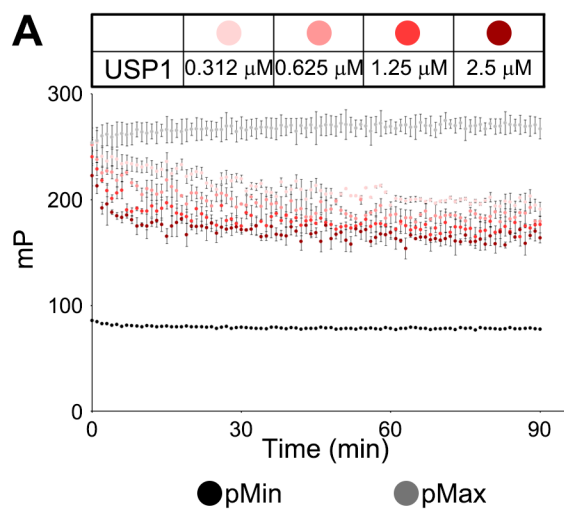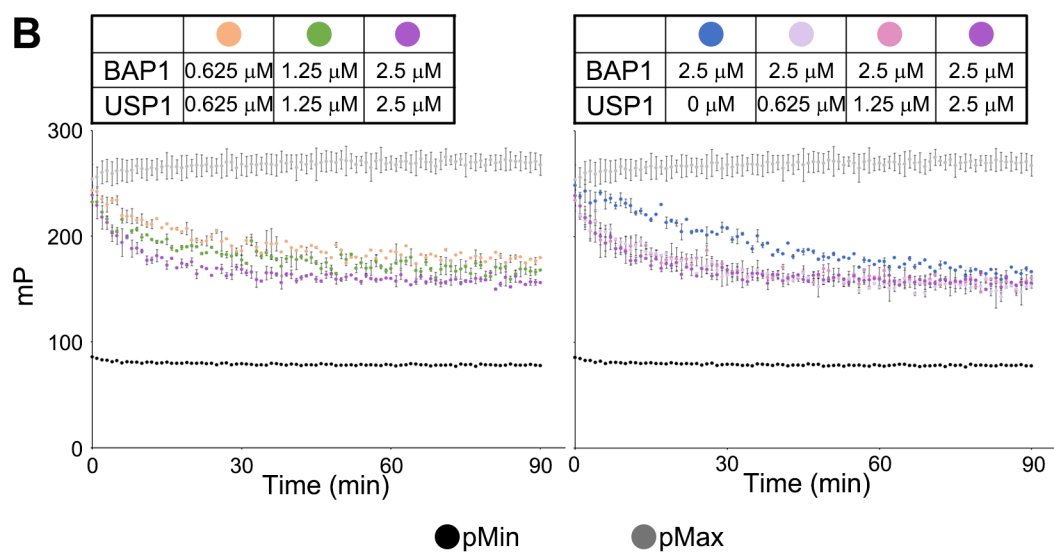

supplementary Figure 2 Suzuki K et al.

Supplement: Supplementary file 3 — Supplemental Figure 2 [file 41419_2026_8818_MOESM3_ESM.pdf]

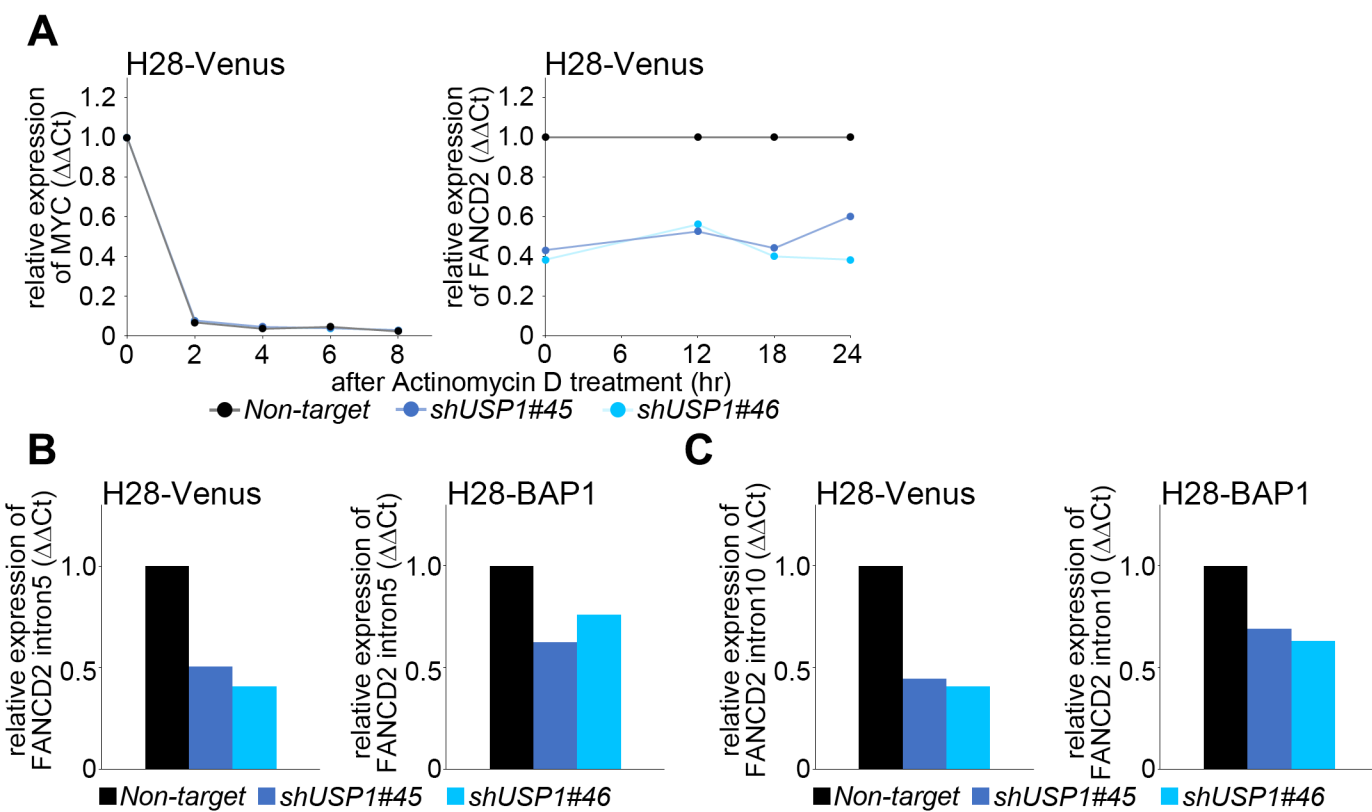

supplementary Figure 3 Suzuki K et al.

Supplement: Supplementary file 4 — Supplemental Figure 3 [file 41419_2026_8818_MOESM4_ESM.pdf]

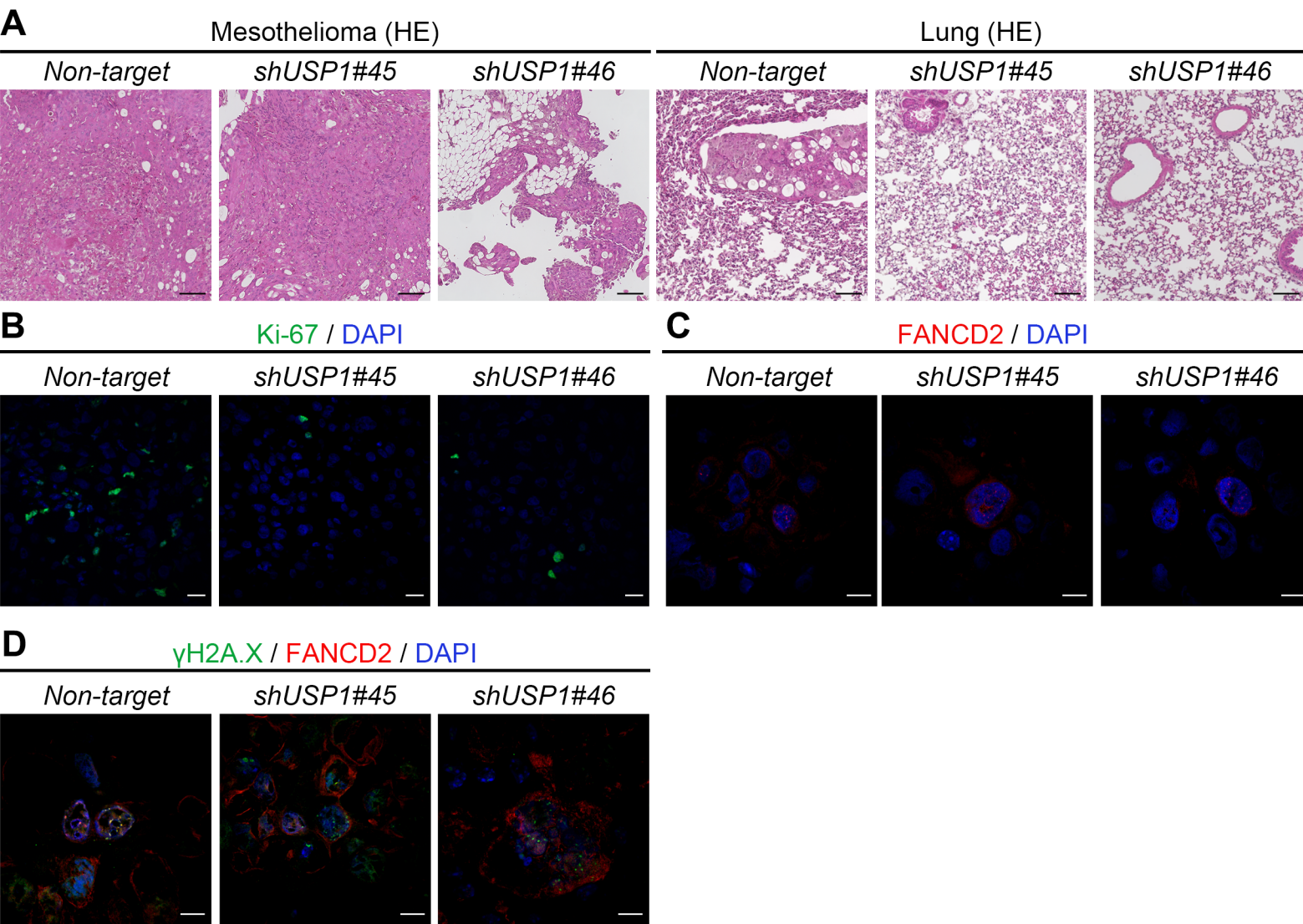

supplementary Figure 4 Suzuki K et al.

Supplement: Supplementary file 5 — Supplemental Figure 4 [file 41419_2026_8818_MOESM5_ESM.pdf]
