# Supplementary material for: BAP1 and USP1 cooperate to regulate FANCD2 stability and cell proliferation in mesothelioma cells
Source: Cell Death Dis. 2026 May 2;17(1):583. doi: 10.1038/s41419-026-08818-7 (PMC13279938; doi:10.1038/s41419-026-08818-7)
Supplement: Supplementary file 6 — Original Data [file 41419_2026_8818_MOESM6_ESM.pdf]

Figure1 B

BAP1 same Exposure uncropped

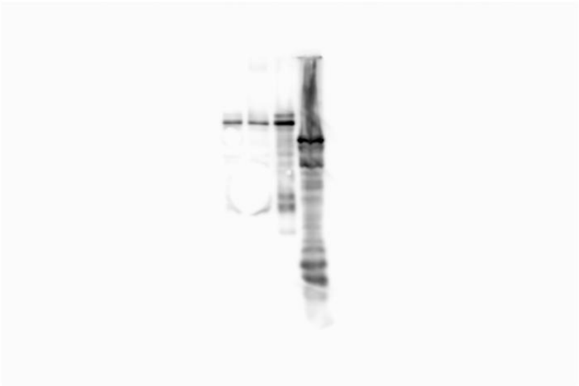

BAP1 with marker

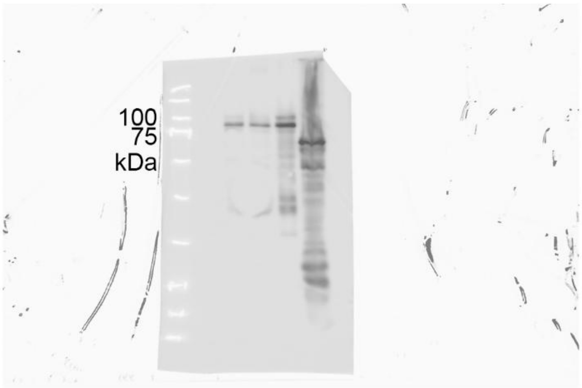

β-actin same Exposure uncropped

β-actin with marker

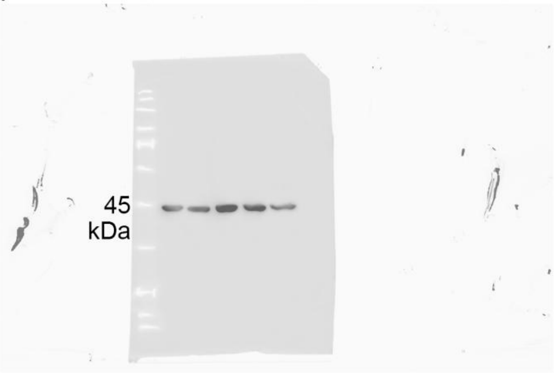

Figure1 C

USP1

ΔCt				
H28 parent	shNT	0.8347049	1.8596206	9.191446
	shUSP1-45	0.1735849	0.3106912	0.8977688
	shUSP1-46	0.1584132	0.3697615	0.7537204
H28 BAP1 O.E.	shNT	1.0221338	1.3785997	6.4084342
	shUSP1-45	0.2104383	0.2227537	0.7623924
	shUSP1-46	0.0461704	0.2279608	0.4640215

ΔΔCt					average	SD
H28 parent	shNT	1	1	1	1	0
	shUSP1-45	0.2079596	0.1670723	0.0976744	0.15756876	0.05575342
	shUSP1-46	0.1897835	0.1988371	0.0820024	0.15687432	0.06499883
H28 BAP1 O.E.	shNT	1	1	1	1	0
	shUSP1-45	0.2058813	0.1615797	0.118967	0.16214267	0.04345988
	shUSP1-46	0.0451706	0.1653568	0.0724079	0.09431176	0.06301599

Figure1 F

USP1

ΔCt						
H2452 parent	shNT	5.771637	3.740628	2.204751	8.394625	2.327994
	shUSP1-45	2.41521	1.343568	1.287408	3.526543	0.644735
	shUSP1-46	4.759012	0.950617	1.576761	3.379479	0.537046
H2452 BAP1 O.E.	shNT	5.866329	3.608265	4.82715		1.69698
	shUSP1-45	1.92999	1.475018			0.676633
	shUSP1-46	4.100038	1.031293	2.576564		0.560422

ΔΔCt								average	SD
H2452 parent	shNT	1	1	1	1	1		1	0
	shUSP1-45	0.418462	0.359183	0.583925	0.420095	0.276949		0.41172264	0.112576064
	shUSP1-46	0.824552	0.254133	0.715165	0.402577	0.23069		0.48542327	0.270664392
H2452 BAP1 O.E.	shNT	1	1	1		1		1	0
	shUSP1-45	0.328994	0.408789			0.398728		0.37883698	0.043456989
	shUSP1-46	0.69891	0.285814	0.533765		0.330246		0.46218393	0.19120407

Figure3 C
USP1

ΔCt					
H2452 parent	shNT	2.56743	3.208185	1.973573	5.632473
	shUSP1-45	0.157697	0.143411	1.186091	1.901649
	shUSP1-46	0.567129	0.215727	2.075358	1.719106
H2452 BAP1 O.E.	shNT	0.767987	0.292453	3.840372	4.472802
	shUSP1-45	0.109621	0.019334	0.375056	0.312287
	shUSP1-46	0.049478	0.071231	0.657661	0.487925

ΔΔCt							average	SD
H2452 parent	shNT	0.889059	1.110941	0.518949	1.481051		1	0.40308686
	shUSP1-45	0.054608	0.049661	0.311881	0.500036		0.229047	0.218254415
	shUSP1-46	0.196388	0.074703	0.545713	0.452037		0.31721	0.218936557
H2452 BAP1 O.E.	shNT	1.448431	0.551569	0.923924	1.076076		1	0.371373868
	shUSP1-45	0.206747	0.036463	0.090232	0.075131		0.102143	0.073319719
	shUSP1-46	0.093316	0.134342	0.158221	0.117386		0.125816	0.027386868

Figure3 D

FANCD2 same Exposure uncropped

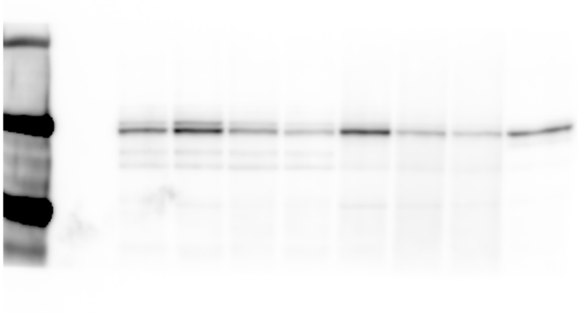

FANCD2 with marker

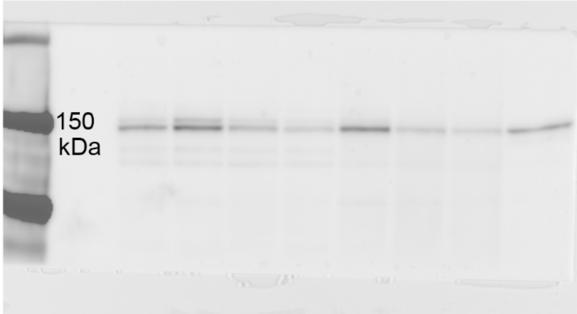

Vinculin same Exposure uncropped

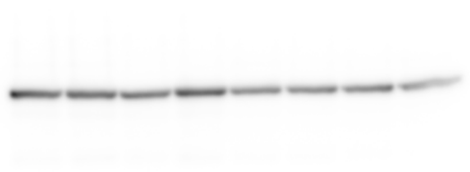

Vinculin with marker

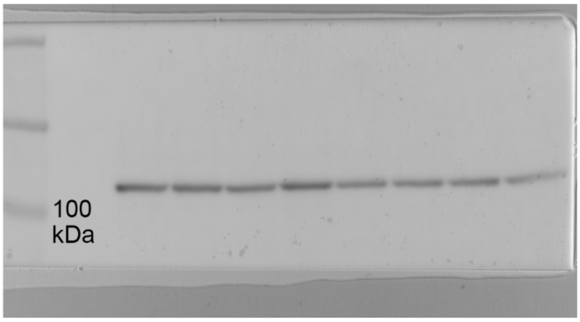

Figure3 D

Ub-FANCD2

H28	shNT	1778.418
	shUSP1#45	885.0893
	shUSP1#46	483.3751
H28-BAP1	shNT	1202.189
	shUSP1#45	667.6512
	shUSP1#46	480.087

H28	shNT	915.6402
	shUSP1#45	510.8936
	shUSP1#46	311.736
H28-BAP1	shNT	639.7589
	shUSP1#45	407.02
	shUSP1#46	344.8496

FANCD2

H28	shNT	3393.72
	shUSP1#45	2102.948
	shUSP1#46	1130.144
H28-BAP1	shNT	4176.978
	shUSP1#45	1799.891
	shUSP1#46	1302.098

H28	shNT	1698.601
	shUSP1#45	1004.074
	shUSP1#46	703.776
H28-BAP1	shNT	2226.716
	shUSP1#45	937.1037
	shUSP1#46	773.49

Ub-FANCD2/(Ub-FANCD2+FANCD2)

H28	shNT	0.343846
	shUSP1#45	0.296211
	shUSP1#46	0.299578
H28-BAP1	shNT	0.22349
	shUSP1#45	0.270573
	shUSP1#46	0.269381

ub-FANCD2/(Ubi-FANCD2+FANCD2)

H28	shNT	0.350251
	shUSP1#45	0.337231
	shUSP1#46	0.306974
H28-BAP1	shNT	0.223187
	shUSP1#45	0.302814
	shUSP1#46	0.308359

Ub-FANCD2/(Ub-FANCD2+FANCD2)

		average	error bar
H28	shNT	0.347048	0.004529
	shUSP1#45	0.316721	0.029005
	shUSP1#46	0.303276	0.00523
H28-BAP1	shNT	0.223338	0.000214
	shUSP1#45	0.286694	0.022798
	shUSP1#46	0.28887	0.027561

Figure5 A

γ H2AXpositive

	H28	H28	H28	H28	H28BAP1	H28BAP1	H28BAP1	H28BAP1
	2022/6/22	2022/7/6	2022/7/13	2022/8/5	2022/6/22	2022/7/6	2022/7/13	2022/8/5
intact-1	56.25	66.66666667	61.76470588	72	34.375	51.5625	49.05660377	44
intact-2	65.90909091	72.72727273	74.28571429	70.58823529	26.31578947	54.38596491		42.85714286
intact-3	70.58823529	57.5	62.74509804	59.375	25	48.71794872	66.12903226	33.33333333
intact-4	62.5	63.88888889	78.37837838	65.71428571	36.66666667	56	68.33333333	38.0952381
intact-5		60	93.93939394	78.57142857	55.88235294		72	34.375
intact-6		67.85714286	87.5					
intact-7		60						
intact-8		65.95744681						
intact-9		60.86956522						
intact-10		69.04761905						
intact-11		89.65517241						
shNT-1	55.55555556	71.875	73.52941176	85.29411765	37.03703704	46.15384615	46.875	51.35135135
shNT-2	73.68421053	58.97435897	74.19354839	75	35.71428571	55.26315789	77.14285714	52.17391304
shNT-3	80	85.29411765	70	80	36.58536585	46.51162791	48.57142857	57.69230769
shNT-4	76.47058824	66.66666667	72.72727273	66.66666667	47.61904762	54.34782609	45	48.14814815
shNT-5	73.68421053	75.86206897	80.64516129	81.25	40	33.89830508	43.90243902	54.54545455
shNT-6		59.375	75	83.33333333		46.42857143	70.58823529	48.27586207
shNT-7		70.96774194				39.39393939		
shNT-8		70.96774194				48.86363636		
shNT-9		58.53658537				50.51546392		
shNT-10		63.88888889						
shNT-11		79.41176471						
shUSP1-1	91.66666667	73.07692308	80	83.33333333	66.66666667	33.94495413	65.2173913	67.64705882
shUSP1-2	85.71428571	83.33333333	80.95238095	85.71428571	47.05882353	49.54954955	76	56.52173913
shUSP1-3	66.66666667	68.42105263	90.47619048	72.22222222	33.33333333	48.31460674	55.55555556	41.93548387
shUSP1-4	88.88888889	68.18181818	70.27027027	91.30434783	29.41176471	54.65116279	52.38095238	58.62068966
shUSP1-5	80	61.53846154	65.95744681	71.875	47.05882353	43.68932039	47.05882353	45.16129032
shUSP1-6	90	82.14285714				44.56521739	73.52941176	53.84615385
shUSP1-7		80				51.94805195		
shUSP1-8		85.18518519				53.33333333		
shUSP1-9		56				57.5		
shUSP1-10		73.07692308				50		
shUSP1-11		63.33333333				58.69565217		

	H28	H28	H28BAP1	H28BAP1
	shNT	shUSP1#45	shNT	shUSP1#45
average	72.81621471	77.38266196	48.56150413	52.25699323
SD	8.124756167	10.09182279	9.779479072	11.20609797

	H28	H28BAP1	H28	H28BAP1
	shNT	shNT	shUSP1#45	shUSP1#45
average	72.81621471	48.56150413	77.38266196	52.25699323
SD	8.124756167	9.779479072	10.09182279	11.20609797

H28shNT	28	72.81621471	66.01166278	8.124756167	1.535434592
H28shUSP1	27	77.38266196	101.8448871	10.09182279	1.942172201
BAP1shNT	26	48.56150413	95.63821092	9.779479072	1.917913639
BAP1shUSP1	28	52.25699323	125.5766318	11.20609797	2.117753457
	109	62.88055782	251.3452684	15.85387235	1.518525566

Tukey-Kramer

H28shNT,H28shUSP1	-4.56645	6.944268499	8.484100461	-1.716727502	2.610654637	3.189544905	
H28shNT,BAP1shNT	24.25471	7.011924765	8.566758924	9.030426703	2.610654637	3.189544905	**
H28shNT,BAP1shUSP1	20.55922	6.880849193	8.406618468	7.800349256	2.610654637	3.189544905	**
H28shUSP1,BAP1shNT	28.82116	7.074169087	8.64280539	10.63617344	2.610654637	3.189544905	**
H28shUSP1,BAP1shUSP1	25.12567	6.944268499	8.484100461	9.4458392	2.610654637	3.189544905	**
BAP1shNT,BAP1shUSP1	-3.69549	7.011924765	8.566758924	-1.375891225	2.610654637	3.189544905	

Figure5 B

FANCD2

	H28				H28BAP1			
	22.06.22	22.07.6	22.07.13	22.08.5	22.06.22	22.07.6	22.07.13	22.08.5
intact-1	68.75	46.15385	67.64706	52	37.5	40.625	66.03774	24
intact-2	56.81818	54.54545	60	64.70588	34.21053	50.87719		32.14286
intact-3	76.47059	50	54.90196	50	30	44.87179	48.3871	28.20513
intact-4	77.5	61.11111	67.56757	51.42857	46.66667	48	60	33.33333
intact-5		57.14286	66.66667	78.57143	58.82353		72	26.5625
intact-6		67.85714	68.75					
intact-7		35						
intact-8		44.68085						
intact-9		32.6087						
intact-10		57.14286						
intact-11		79.31034						
shNT-1	66.66667	65.625	67.64706	64.70588	37.03704	38.46154	40.625	32.43243
shNT-2	84.21053	64.10256	70.96774	62.5	42.85714	42.10526	48.57143	34.78261
shNT-3	70	76.47059	62.5	80	31.70732	44.18605	45.71429	57.69231
shNT-4	76.47059	66.66667	69.69697	55.55556	61.90476	39.13043	42.5	40.74074
shNT-5	68.42105	68.96552	67.74194	56.25	50	35.59322	63.41463	50
shNT-6		59.375	79.16667	61.11111		33.92857	61.76471	41.37931
shNT-7		61.29032				36.36364		
shNT-8		54.83871				42.04545		
shNT-9		46.34146				35.05155		
shNT-10		55.55556						
shNT-11		73.52941						
shUSP1-1	100	26.92308	75	55.55556	66.66667	22.01835	60.86957	29.41176
shUSP1-2	92.85714	55.55556	61.90476	71.42857	70.58824	31.53153	48	43.47826
shUSP1-3	88.88889	57.89474	76.19048	55.55556	25	39.32584	55.55556	29.03226
shUSP1-4	72.22222	43.18182	59.45946	60.86957	35.29412	37.2093	52.38095	48.27586
shUSP1-5	80	30.76923	53.19149	62.5	47.05882	33.00971	58.82353	35.48387
shUSP1-6	90	57.14286				33.69565	76.47059	30.76923
shUSP1-7		40				36.36364		
shUSP1-8		66.66667				41.11111		
shUSP1-9		40				43.75		
shUSP1-10		53.84615				39.28571		
shUSP1-11		50				41.30435		

	H28	H28	H28BAP1	H28BAP1
	shNT	shUSP1#45	shNT	shUSP1#45
average	66.2990198	62.1334735	43.4611317	43.2773028
SD	8.5942132	18.3985923	9.19127287	13.8289089

	H28	H28BAP1	H28	H28BAP1
	shNT	shNT	shUSP1#45	shUSP1#45
average	66.2990198	43.4611317	62.1334735	43.2773028
SD	8.5942132	9.19127287	18.3985923	13.8289089

H28shNT	28	66.29902	73.8605	8.594213	1.624154
H28shUSP1	27	62.13347	338.5082	18.39859	3.540811
BAP1shNT	26	43.46113	84.4795	9.191273	1.802557
BAP1shUSP1	28	43.2773	191.2387	13.82891	2.613418
	109	53.90578	279.6166	16.72174	1.601652

tukey-kramer

H28shNT,H28shUSP1	4.16554631	9.23772153	11.286107	1.177217	2.610655	3.189545	
H28shNT,BAP1shNT	22.8378881	9.32772234	11.3960647	6.39189679	2.610655	3.189545	**
H28shNT,BAP1shUSP1	23.0217171	9.15335701	11.1830354	6.56608852	2.610655	3.189545	**
H28shUSP1,BAP1shNT	18.6723418	9.41052382	11.4972267	5.1800555	2.610655	3.189545	**
H28shUSP1,BAP1shUSP1	18.8561708	9.23772153	11.286107	5.32890599	2.610655	3.189545	**
BAP1shNT,BAP1shUSP1	0.18382898	9.32772234	11.3960647	0.05145029	2.610655	3.189545	

Figure5 C

	H28		H28 BAP1		H28	H28BAP1	H28	H28BAP1	H28	H28BAP1
intact1-1	15	42	21	55	35.71429	38.18182	36.81092	33.20261	5.837856	4.102299
intact1-2			11	59		18.64407				
intact1-3			20	48		41.66667				
intact2-1			26	54		48.14815				
intact2-2			19	52		36.53846				
intact2-3			10	46		21.73913				
intact3										
intact4-1	18	50			36					
intact4-2	26	50			52					
intact5-1	12	51	22	80	23.52941	27.5				
NT1	17	65	15	42	26.15385	35.71429	35.77923	33.68094	5.650447	2.744554
NT2										
NT3-1	17	71	19	52	23.94366	36.53846				
NT3-2	11	37			29.72973					
NT4-1	27	50	11	48	54	22.91667				
NT4-2	12	43			27.90698					
NT5-1	27	51	14	40	52.94118	35				
NT5-2			13	34		38.23529				
shUSP1 1-	14	55			25.45455		19.93377	36.61775	3.548636	8.428333
shUSP1 1-	10	40			25					
shUSP1 2-	4	43			9.302326					
shUSP1 3-	16	49	5	36	32.65306	13.88889				
shUSP1 3-	12	48			25					
shUSP1 4	8	58			13.7931					
shUSP1 5	4	48			8.333333					
shUSP1 6-1			11	53		20.75472				
shUSP1 6-2			17	43		39.53488				
shUSP1 7-1			21	40		52.5				
shUSP1 7-2			22	39		56.41026				

	H28	H28BAP1	H28	H28BAP1
	shNT	shNT	shUSP1#45	shUSP1#45
average	35.77923185	33.68094161	19.93376701	36.6177492
SD	13.84071079	6.137009965	9.388807438	18.8463245

	H28	H28	H28BAP1	H28BAP1
	shNT	shUSP1#45	shNT	shUSP1#45
average	35.77923185	19.93376701	33.68094161	36.6177492
SD	13.84071079	9.388807438	6.137009965	18.8463245

H28shNT	6	35.77923	191.5653	13.84071	5.650447
H28shUSP1	7	19.93377	88.14971	9.388807	3.548636
BAP1shNT	5	33.68094	37.66289	6.13701	2.744554
BAP1shUSP1	5	36.61775	355.1839	18.84632	8.428333
	23	30.68284	192.9004	13.88886	2.896027

H28shNT,H28shUSP1	15.84546	19.84695	25.22368694	2.244957186	2.811880826	3.573646961
H28shNT,BAP1shNT	2.09829	21.60143	27.45347043	0.273136635	2.811880826	3.573646961
H28shNT,BAP1shUSP1	-0.83852	21.60143	27.45347043	-0.109150681	2.811880826	3.573646961
H28shUSP1,BAP1shNT	-13.7472	20.88831	26.54716429	-1.850576137	2.811880826	3.573646961
H28shUSP1,BAP1shUSP1	-16.684	20.88831	26.54716429	-2.24591454	2.811880826	3.573646961
BAP1shNT,BAP1shUSP1	-2.93681	22.56196	28.67421468	-0.36601224	2.811880826	3.573646961

Figure6 A

ΔCt

H226 parent	shNT	2.384589	1.4648	9.308569	4.447911
	shUSP1-45	0.481227	0.45382	3.612236	1.841494
	shUSP1-46	0.717275	0.614187	3.870694	2.14711
H226 BAP1 O.E.	shNT	2.376799	1.503429	8.118213	
	shUSP1-45	0.436556	0.403463	1.638223	
	shUSP1-46	0.345145	0.523543	2.849419	

ΔΔCt

						average	SD
H226 parent	shNT	1	1	1	1	1	0
	shUSP1-45	0.201807	0.309817	0.388055	0.414013	0.32842313	0.09532305
	shUSP1-46	0.300796	0.419297	0.41582	0.482723	0.4046593	0.07576372
H226 BAP1 O.E.	shNT	1	1	1		1	0
	shUSP1-45	0.183674	0.268362	0.201796		0.21794394	0.04459338
	shUSP1-46	0.145214	0.348232	0.350991		0.28147918	0.11801709

Figure6 B

mouse Luminescence uncropped data

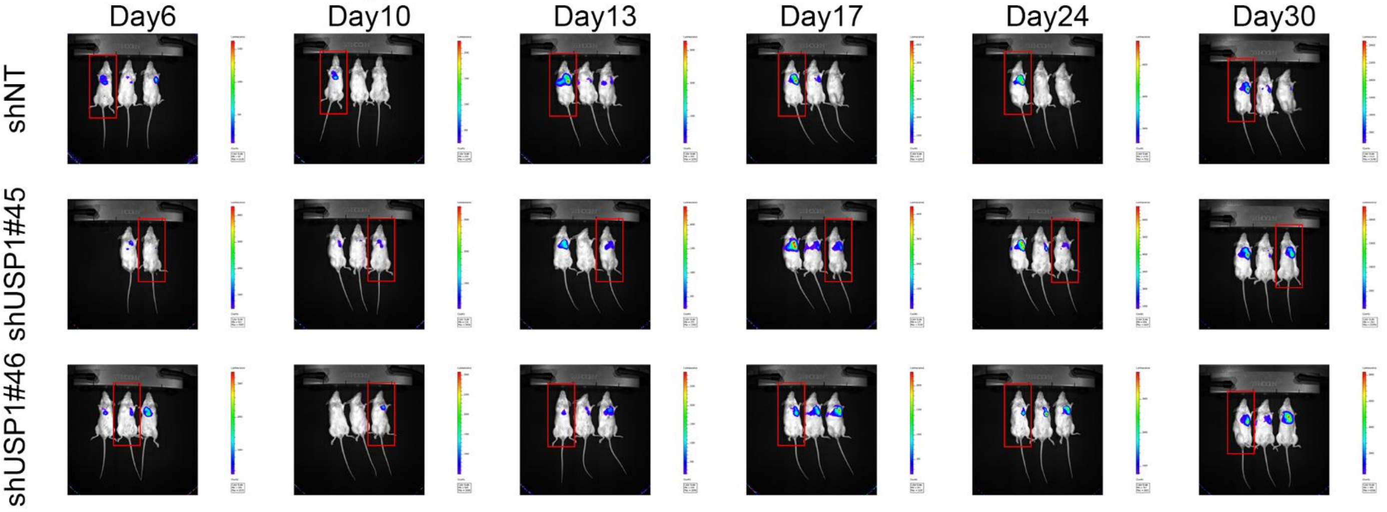

Figure6 B

		days after transplantation					
cell	shRNA	6	10	13	17	24	30
H226	shNT	152100	421700	483300	1130000	1554000	8180000
		52330	750400	563800	899800	1260000	2919000
		106500	498900	531600	977100	1329000	8737000
		121400	173200	1270000	2181000	3033000	7000000
		187500	158300	1544000	1259000	4504000	9908000
	shUSP1#45	65330	83880	35720	199900	118100	617000
		56650	66670	62520	160700	274700	668600
		92600	95290	484800	700600	1201000	1880000
		162900	98470	77840	237600	415300	304200
	shUSP1#46	74590	104000	46640	275600	377400	1056000
		107200	249800	141200	223900	350600	2048000
		63920	184800	682300	259900	641900	922400
		1640	1762	2820	4588	631000	3498000

			days after transplantation					
cell	shRNA		6	10	13	17	24	30
H226	shNT	average	123966	400500	878540	1289380	2336000	7348800
		SD	50674.09	246418.4	492879.6	517349.6	1411505	2688507
		SEM	22662.14	110201.7	220422.5	231365.8	631244.1	1202337
	shUSP1#45	average	94370	86077.5	165220	324700	502275	867450
		SD	48185.8	14375.03	213763.2	252559.1	481373.8	693968
		SEM	24092.9	7187.514	106881.6	126279.5	240686.9	346984
	shUSP1#46	average	61837.5	135090.5	218240	190997	500225	1881100
		SD	44152.99	107039.6	314716.4	126143.1	157741.9	1189134
		SEM	19745.82	47869.55	140745.4	56412.89	70544.33	531796.8

Supplementary Figure1 A

BAP1 same Exposure

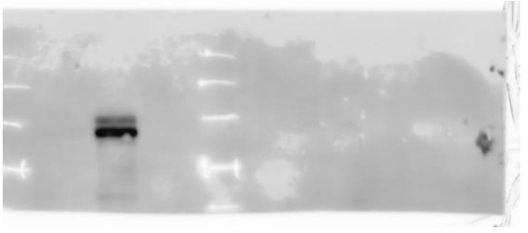

BAP1 with marker uncropped

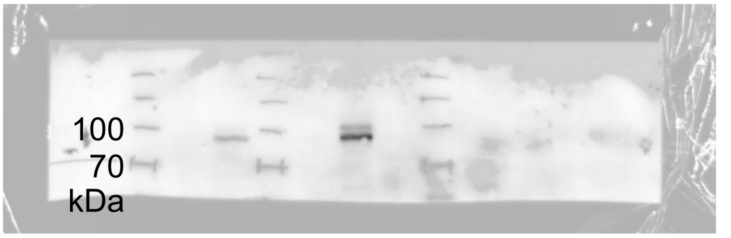

β-actin same Exposure

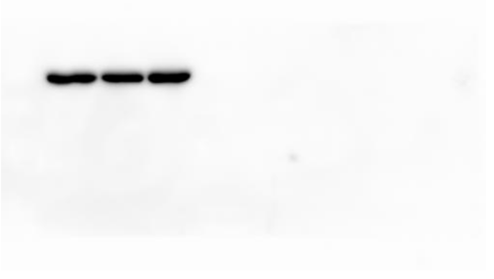

β-actin with marker uncropped

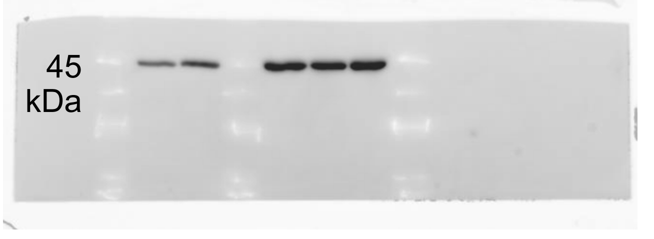

Supplementary Figure3 A

H28_FANCD2

		shNT	shSUP1#45	shUSP1#46
		ΔΔCt(/shNT)		
time	0	1	0.430480395	0.382244088
	12	1	0.524945973	0.560614957
	18	1	0.44071286	0.399647055
	24	1	0.600353808	0.381770148

H28_Myc_0h

		shNT	shSUP1#45	shUSP1#46
		ΔCt(/GAPDH)		
time	0	1	1	1
	2	0.068546112	0.078753402	0.07599383
	4	0.037444478	0.047514358	0.041447358
	6	0.048002085	0.041494279	0.039357504
	8	0.024518937	0.029266195	0.029572793

H28BAP1_FANCD2

		shNT	shSUP1#45	shUSP1#46
		ΔΔCt(/shNT)		
time	0	1	0.95513212	0.6868632
	12	1	0.759932765	1.224908916
	18	1	0.696638825	0.735233941
	24	1	0.673191302	0.754294183

H28BAP1_Myc_0h

		shNT	shSUP1#45	shUSP1#46
		ΔCt(/GAPDH)		
time	0	1	1	1
	2	0.161621225	0.041436657	0.052253331
	4	0.035867202	0.176078275	0.160746455
	6	0.027614341	0.033021844	0.029441642
	8	0.01703087	0.015536006	0.016454264

Supplementary Figure3 B

		H28			H28BAP1		
		shNT	shUSP1#45	shUSP1#46	shNT	shUSP1#45	shUSP1#46
ΔCT	intron5	0.462	0.235	0.162	0.556	0.322	0.410
		0.390	0.196	0.186	0.521	0.352	0.411
	average	0.426	0.215	0.174	0.539	0.337	0.410
ΔΔCT	average	1	0.505737205	0.408799898	1	0.626265095	0.761589005

Supplementary Figure3 C

		H28			H28BAP1		
		shNT	shUSP1#45	shUSP1#46	shNT	shUSP1#45	shUSP1#46
ΔCT	intron10	0.301	0.121	0.131	0.269	0.190	0.177
		0.285	0.139	0.108	0.279	0.189	0.170
	average	0.293	0.130	0.120	0.274	0.189	0.173
ΔΔCT	average	1	0.444781341	0.408437626	1	0.691371983	0.632237748

		H28			H28BAP1		
		shNT	shUSP1#45	shUSP1#46	shNT	shUSP1#45	shUSP1#46
ΔCT	intron5	0.462	0.235	0.162	0.556	0.322	0.410
		0.390	0.196	0.186	0.521	0.352	0.411
	average	0.426	0.215	0.174	0.539	0.337	0.410
ΔΔCT	average	1	0.505737205	0.408799898	1	0.626265095	0.761589005

		H28			H28BAP1		
		shNT	shUSP1#45	shUSP1#46	shNT	shUSP1#45	shUSP1#46
ΔCT	intron10	0.301	0.121	0.131	0.269	0.190	0.177
		0.285	0.139	0.108	0.279	0.189	0.170
	average	0.293	0.130	0.120	0.274	0.189	0.173
ΔΔCT	average	1	0.444781341	0.408437626	1	0.691371983	0.632237748
